# The *d* Orbital Multi Pattern Occupancy in a Partially Filled d Shell: The KFeF_3_ Perovskite as a Test Case

**DOI:** 10.3390/ma16041532

**Published:** 2023-02-12

**Authors:** Fabien Pascale, Sami Mustapha, Philippe D’Arco, Roberto Dovesi

**Affiliations:** 1 Laboratoire de Physique et Chimie Théoriques, Université de Lorraine, CNRS, UMR 7019, F-54506 Vandoeuvre-lès-Nancy, France; 2 Institut de Mathématiques de Jussieu, Sorbonne Université, UMR 7586, F-75005 Paris, France; 3 Institut des Sciences de la Terre, Sorbonne Université, CNRS-INSU, ISTeP UMR 7193, F-75005 Paris, France; 4 Dipartimento di Chimica, Università di Torino, Via P. Giuria 5, 10125 Torino, Italy

**Keywords:** KFeF_3_ perovskite, Jahn–Teller effect, cubic or tetragonal, orientation of the unit cell, *d* occupancy, simulation, Gaussian type basis set, B3LYP, PBE0, HSE06, Hartree–Fock

## Abstract

The occupancy of the d shell in KFeF${}_{3}$ is $t_{2g}^{4}$e${}_{g}^{2}$, with five α and one β electrons. The Jahn–Teller lift of degeneracy in the $t_{2g}$ sub-shell produces a tetragonal relaxation of the unit cell (4.09 vs. 4.22 Å, B3LYP result) not observed experimentally. In order to understand the origin of this apparent contradiction, we explored, with a 2 × 2 × 2 supercell (40 atoms per cell), all possible local structures in which contiguous Fe atoms have a different occupancy of the $t_{2g}$ orbitals with the minority spin electron. A total of 6561 configurations (with occupancies from (8,0,0) to (3,2,2) of the 3 $t_{2g}$ orbitals of the 8 Fe atoms) have been explored, with energies in many cases lower (by up to 1550 μE${}_{h}$ per 2 Fe atoms) than the one of the fully ordered case, both for the ferromagnetic and the anti-ferromagnetic solutions. The results confirm that the orientation of the β d electron of Fe influences the electrostatics (more efficient relative orientation of the Fe quadrupoles of the d shell) of the system, but not the magnetic interactions. Three hybrid functionals, B3LYP, PBE0, and HSE06, provide very similar results.

## 1. Introduction

Only in a few cases, the ABX${}_{3}$ perovskites maintain the cubic structure. There are at least three mechanisms that produce a symmetry lowering, and, possibly, an increase of the size of the unit cell, from 1 to 2 or 4 or 8 formula units (f.u.). The most common one has been described about 50 years ago by Glazer [1,2], who investigated all possible structures that can be obtained from the aristotype cubic structure by tilting the BX${}_{6}$ octahedra with respect to one, two, or three Cartesian directions, and assuming periods not longer than two octahedra. In general, the octahedra remain regular during the tilting. KMnF${}_{3}$ is an example of compound belonging to this class of perovskites; the many phase transitions it is passing through as a function of the temperature are still a matter of debate [3].

In the second case the transition metal (TM) at the center of the octahedron moves (say vertically), breaking the symmetry (the inversion center is lost), and this activates the ferroelectricity of the compound. A prototype of this family is KNbO${}_{3}$.

The third case involves the Jahn–Teller effect [4,5,6] (for a very recent discussion of the effect, see Reference [7], and the many references therein), that is active when the $t_{2g}$ or $e_{g}$ subshells are partially occupied. Reducing the symmetry allows to lift the degeneracy of the $t_{2g}$ or e${}_{g}$ subshells of the aristotype cubic structure, with a consequent energy gain. It is this third mechanism that will be discussed in the present study.

When looking at the series of the 11 KMF${}_{3}$ perovskites, from Ca to Zn, we would expect that Ca, V, Mn, Ni, and Zn might be involved in the first class (octahedron rotation). Sc, Ti, Fe, and Co are expected to undergo a Jahn–Teller deformation, as the $t_{2g}$ subshell is not filled or half-filled, as it contains 1, 2, 6, and 7 electrons (instead of 3 or 6). Cr and Cu contain 4 and 9 electrons in the *d* shell, and then the Jahn–Teller deformation is expected to be larger, as the e${}_{g}$ orbitals point in the direction of the F first neighbors. And actually KCrF${}_{3}$ and KCuF${}_{3}$ are known to be tetragonal, with a strong deformation of the octahedron: for the Cr compound [8], the in-plane Cr-F distances are 1.97 and 2.01 Å, and the ones along *c* are 2.29 Å, with a percentage difference of nearly 10%. In the KCuF${}_{3}$ case, the numbers [9] are 1.89, 1.96, and 2.25 Å. These two systems become cubic at high temperature: KCrF${}_{3}$ at 973 K, as reported in Ref. [8]; KCuF${}_{3}$ at 800 K (see Ref. [10]).

In the Fe, Co, Sc, and Ti cases, the situation is different, as the $t_{2g}$ orbitals point along the main diagonals of the cube, in between two F${}^{-}$ ions, so that the repulsion is much smaller than for the e${}_{g}$ (Cr, Cu) cases; as a consequence the Jahn–Teller deformation of the cell and the related energy gain is certainly smaller.

The question is then: *is the deformation visible at sufficiently low T?* The structure of the Fe and Co compounds has been investigated in a series of papers [11,12,13], all dating back to the 1960s. At room temperature (T) the system is in all cases reported as cubic. The possibility of a lower symmetry is then investigated at low T:

Knox [11] performed single crystal X-ray measurements. For the Fe compound, in its Table I, under the column *predicted distortion*, it is indicated *small or 0*, and under the column *observed distortion* appears 0; in the KCoF${}_{3}$ case, both the predicted and observed distortions are null. The reported cubic lattice parameters are 4.120 and 4.071 for the Fe and Co compounds, respectively.

Also, Okazaki et al. [12] performed single crystal X-ray determinations. They observe that *below 78 K the lattice symmetry of KFeF${}_{3}$ is rhombohedral (α< 90${}^{\circ }$), and the one of KCoF${}_{3}$ is tetragonal (a > c)*; the cell parameters are *a* = 4.108 Å and α = 89${}^{\circ }$51${}^{\prime }$ for the Fe compound, and *a* = 4.057 Å, and *c* = 4.049 Å for KCoF${}_{3}$. Note, however, that the proposed deformation for KFeF${}_{3}$ is extremely small (9 primes of a degree). In order to quantify this deviation from 90${}^{\circ }$ we performed two calculations, with very high accuracy, imposing the proposed rhombohedral space group with 90${}^{\circ }$ and 89${}^{\circ }$51${}^{\prime }$. The energy difference is smaller than 1·10${}^{-7}$ E${}_{h}$ (less than 1 K), that is, completely negligible. So 89${}^{\circ }$51${}^{\prime }$ seems more the result of a pure statistical averaging between various measurements than a clear evidence of a rhombohedral deformation. There is a second point that should be underlined: on the basis of general considerations. KFeF${}_{3}$ and KCoF${}_{3}$ should behave in a very similar way, according to the electron-hole symmetry (one β electron in KFeF${}_{3}$, one β hole in KCoF${}_{3}$ in the $t_{2g}$ subshell). Okazaki ’s results, on the contrary, propose two different space groups for the two systems. We guess that the difference is generated by the different samples used by Okazaki and collaborators, or simply by the fact that the two sets of experiments have been performed not exactly in the same conditions.

Finally, Scatturin et al. [13] performed neutron diffraction experiments at low temperature (4.2 K). They propose the two systems as cubic, with lattice paramer *a* = 4.042 (4.088) Å for KFeF${}_{3}$ (KCoF${}_{3}$). In summary, There is no experimental evidence that KFeF${}_{3}$ and KCoF${}_{3}$ are not cubic at low and room temperature.

Also, from the side of the simulation, there is no documentation of the distortion of the unit cell. The exception is the recent (2019) paper by Varignon et al. [14], documenting that KFeF${}_{3}$, KCoF${}_{3}$ and other systems are Jahn–Teller distorted; they do not report, however, their optimized geometry, and then the amount of distortion (The octahedron remains regular? How large is the tilting?).

The present manuscript investigates the reasons for this apparent contradiction between the results of the quantum mechanical simulation for KFeF${}_{3}$, that provides a relatively large tetragonal deformation of the cell (3%), and the experimental observations. Here we explore a mechanism, according to which the expected tetragonal distortion is *lost* in a supercell in which the doubly occupied $t_{2g}$ orbital is not the same in contiguous sites, but alternates so as to optimize the electrostatic of the system.

In a supercell containing 8 KFeF${}_{3}$ units, obtained by expanding the aristotypical tetragonal primitive cell by two along the three primary crystallographic directions, and indicated as S222, all symmetry independent configurations (they are 6561) or patterns corresponding to all possible *compositions* are built and optimized, and the energies compared with the one of the fully ordered cell.

These *compositions* are labelled using integer triplets $\left(n^{yz},n^{xz},n^{xy}\right)$, where $n^{uv}$ indicates in how many Fe atoms the β electron occupies the d${}_{uv}$ orbital. Obviously, $n^{yz}+n^{xz}+n^{xy}=8$. The energies are compared with the one of the (0,0,8) configuration, referred to as the *fully ordered* one. The full set of calculations is repeated for the ferromagnetic (FM) and the anti-ferromagnetic (AFM) solutions.

The paper is structured as follows:

In Section 2 the computational conditions are defined. In Section 3, the results obtained with the B3LYP functional are presented, followed by some conclusions. In the Appendix A, tables and figures, complementary to the ones reported in the main text of the manuscript, are reported:

(i) PBE0 and HSE06 results, very close to the B3LYP ones.
(ii) Hartree–Fock (HF) results, qualitatively similar to the B3LYP ones.
(iii) Technical details and results of many inner checks of the accuracy of the obtained results: as 6561 total energies are compared, spanning a relatively narrow energy range, and obtained by optimizing a cell containing 40 atoms, it is essential to show that each one of these energies is determined with an accuracy such that the differences remain meaningful.

## 2. Materials and Methods

The FM and AFM solutions of KFeF${}_{3}$ have been evaluated by using the full range B3LYP [15,16] and PBE0 [17], and the range separated HSE06 [18,19] hybrid functionals, as well as the Hartree–Fock Hamiltonian. An allelectron Gaussian type basis set and the CRYSTAL code [20,21,22] have been adopted. The triple zeta type 7-311G, 8-6-411(41d)G and 8-6-511G contractions, consisting of 13, 27, and 17 Atomic Orbitals (AO) for F, Fe, and K, respectively, are similar to the ones used in our previous study on this compound [23]. Exponents and coefficients of the contractions have, however, been optimized; the basis sets are given in Table A1 and Table A2.

The Coulomb and Hartree–Fock exchange series are controlled by five parameters [24] that were set to T${}_{1}$ = T${}_{2}$ = T${}_{3}$ = T${}_{4}$ and T${}_{5}$ = 2·T${}_{1}$, with T${}_{1}$ = 10 (for a complete description of the role of these parameters, see also Refs. [25,26]); these values are required for an accurate evaluation of the small differences (in energy and geometry) between the various configurations of the system, ranging between $10^{-3}$ and $10^{-5}$ E${}_{h}$. As regards the DFT exchange-correlation contribution to the Fock matrix, it was evaluated by numerical integration over the unit cell volume. Radial and angular points for the integration grid were generated through a Gauss–Legendre radial quadrature and Lebedev two-dimensional angular point distributions. In the present work, a pruned grid with 99 radial and 1454 angular points was used (see XXLGRID keyword in the CRYSTAL manual [24]).

In the geometry optimization, the BFSG scheme [27,28,29,30,31] has been adopted.

The process stops when the root-mean-square (RMS) and the absolute value of the largest component of both the gradients and the estimated displacements are smaller than the corresponding thresholds. The default values for the four parameters controlling the process are:

1. TOLDEG (rms of the gradient components) 3·10${}^{-4}$ E${}_{h}$/Bohr.
2. TOLDEGM (maximum value of one gradient component) = 1.5 TOLDEG.
3. TOLDEX (rms of the displacements) 1.2·10${}^{-3}$ Bohr.
4. TOLDEXM (maximum value of the displacement on a single coordinate) = 1.5 TOLDEX.

When these four conditions are simultaneously satisfied, the optimization stops. For the present investigation, in view of the small energy differences between the various configurations, the default values reported above have been divided by 10.

### 2.1. Considered Cells

**Figure 1:**
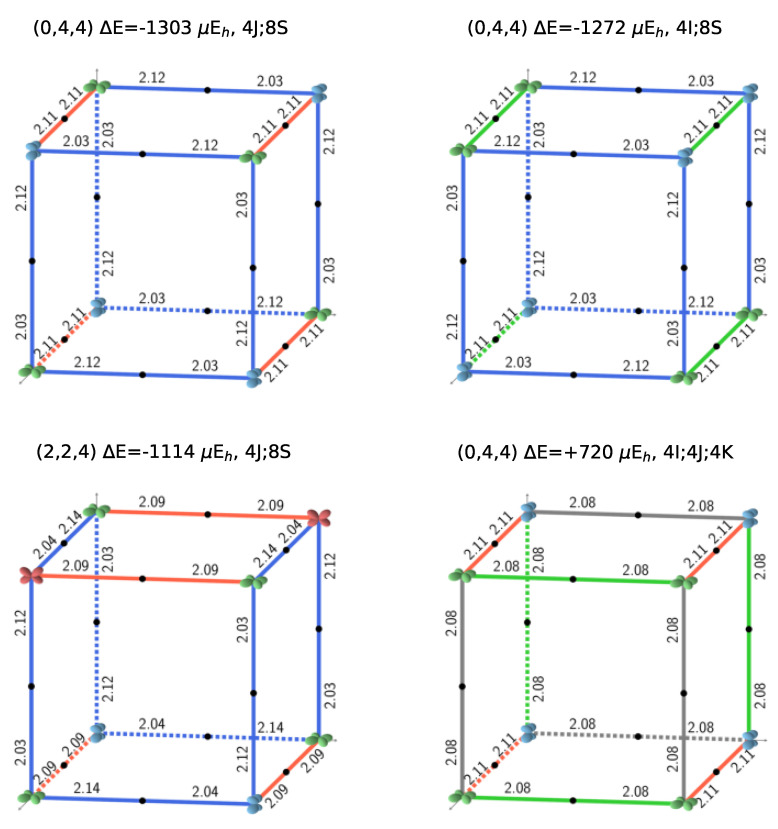
The three most stable and the least stable (**bottom right**) SICs. B3LYP AFM energies refer to two formula units. The doubly occupied *d* orbital on Fe is shown; red, blue, and green indicate $d_{yz}$, $d_{xz}$ and $d_{xy}$, respectively. The relative orientation of the *d* orbitals or interactions are indicated as colored edges: blue: S, green: I, red: J, and gray: K. Half the number of interactions in the corresponding S222 cell present in each case is summarized above the *cube* (for example 4J;8S for the 12 edges of the top left cube), together with the CEC (class of equivalent composition), ΔE and ΔV. The corresponding FM B3LYP energies are −1549, −1428, −1373, and +593 μE${}_{h}$, respectively. The four configurations can be described with a smaller unit cell, with space groups Fmmm (N. 69, orthorhombic, 2 f.u.), Pnma (N. 62, orthorhombic, 4 f.u.), P42/mnm (N. 136, tetragonal, 4 f.u.), and Cmmm (N. 65, orthorhombic, 4 f.u.). The identification of the space group has been performed by using the spglib code [32].

Two cells were considered. The first one, referred to as the *reference case, or fully ordered case* (REF), contains one formula unit with a single transition metal atom, and then 5 atoms overall. It corresponds to the FM state with the cubic system Pm$\bar{3}$m (221). The single β*d* electron is localized in the $d_{xy}$ orbital, that is then doubly occupied. The two other $t_{2g}$*d* orbitals are singly occupied. Obviously, $d_{xy}$ can be replaced by $d_{xz}$ or $d_{yz}$. The Jahn–Teller effect implies that REF is tetragonal. For the AFM solution, REF is doubled. Combining various *d* occupancies in contiguous lattice positions requires larger cells. A 40-atoms supercell, obtained by expanding the unit cell of the aristotype by 2 × 2 × 2 has been used. It contains 8 metal atoms (see Figure 1) and is referred to as S222 with the orthorhombic space group Pmmm (47).

For simplicity, most of the presented results will refer to the FM solution only. For the B3LYP case, however, also the AFM results will be presented and compared with the FM ones.

The AFM solutions will be shown to follow the same path as the FM ones, as the energy differences between the various configurations are due to electrostatics, that is not altered by the FM-AFM transition. In the following, energies and volumes will refer to 2 f.u. (10 atoms).

### 2.2. Symmetry Independent Classes

In a cell containing *n* Fe atoms, the set of distinct *d* occupancies is $\left|S\right|=3^{n}$; in S222, n=8, so |S|=6561. They correspond to 10 different composition classes, (0,0,8), (0,1,7), (0,2,6), (0,3,5), (0,4,4), (1,1,6), (1,2,5), (1,3,4), (2,2,4), and (2,3,3). Permutations of the *d* orbitals naturally dispatch the set of configurations in classes of equivalent compositions (CEC). For example (1,2,5) ≡ (1,5,2) ≡ (2,5,1) ≡ (2,1,5) ≡ (5,1,2) ≡ (5,2,1) or (4,4,0) ≡ (4,0,4) ≡ (0,4,4). As discussed by Mustapha et al. (see Refs. [33,34] and references therein), the symmetry of the aristotype can reduce significantly the number of configurations to be calculated. The symmetry group of the aristotype (and possibly other groups) partitions naturally the set of configurations in symmetry independent classes (SIC). The number of configurations belonging to a SIC is its multiplicity, *M*. The *M* configurations belonging to the same SIC have the same energy and volume, and their vectorial properties are identical within a change of basis.

So the determination of the number of SICs can reduce the resource requirement because only one configuration must be calculated in the *M* set. The determination of the number of SICs, and their enumeration, has been discussed by several authors (see Refs. [33,34,35,36,37,38,39]) in the case of solid solutions, where various atomic species occupy a given set of positions (*D*) within the cell. In that case, atoms considered as colors are isotropic, so they do not possess directional properties as the d orbitals do. This makes the present case different with respect to the solid solution case. Obviously these differences apply to cases with partial *f* or *g* orbital occupancy as well. Mathematical details of the method will be given elsewhere [40] (for additional information, see also Ref. [33]). The list of the 162 SICs and some of their properties are summarized in Table A3 and Table A4. The results of this analysis is that the 6561 configurations belong to 162 SICs. Then, for the purposes of the present study, in which we are interested mainly in the total energy and volume of each configuration, SCF calculations and geometry optimizations can be limited to the 162 SICs.

Each representative of a SIC will be identified in the following by the 3 integers, (n${}^{xy}$,n${}^{xz}$,n${}^{yz})$, with the condition that n${}^{xy}$+n${}^{xz}$+n${}^{yz}$=8.

## 3. Results

KFeF${}_{3}$ is a very ionic compound, with net charges, as obtained from a Mulliken analysis, very close to the formal +1, +2, and −1 charges on K, Fe, and F, respectively, as Table 1 shows. For the four functionals here considered, two columns are reported: the first one (REF, for *reference case*) provides the net charges of the primitive cell containing 5 atoms. The second column (SOL) shows the maximum absolute difference for this net charge among the 8 positions of all the S222 explored configurations. The SOL columns document that in the various configurations the net charges are extremely close to the ones of the REF case. For Fe, the oscillation is always smaller than 0.012 |e|; for F and K it is even smaller. So we can conclude that the net charges remain *nearly* constant in all sites of the full set of explored configurations.

The occupancy of the *d* shell on Fe is $t_{2g}^{4}$$e_{g}^{2}$, with five α electrons and only one β electron in the $t_{2g}$ sub-shell, a typical case in which the Jahn–Teller theorem applies. Table 1 reports (line 5) the number of electrons in the doubly occupied $t_{2g}$ orbital, that in our REF convention is $d_{xy}$.

**Figure 2:**
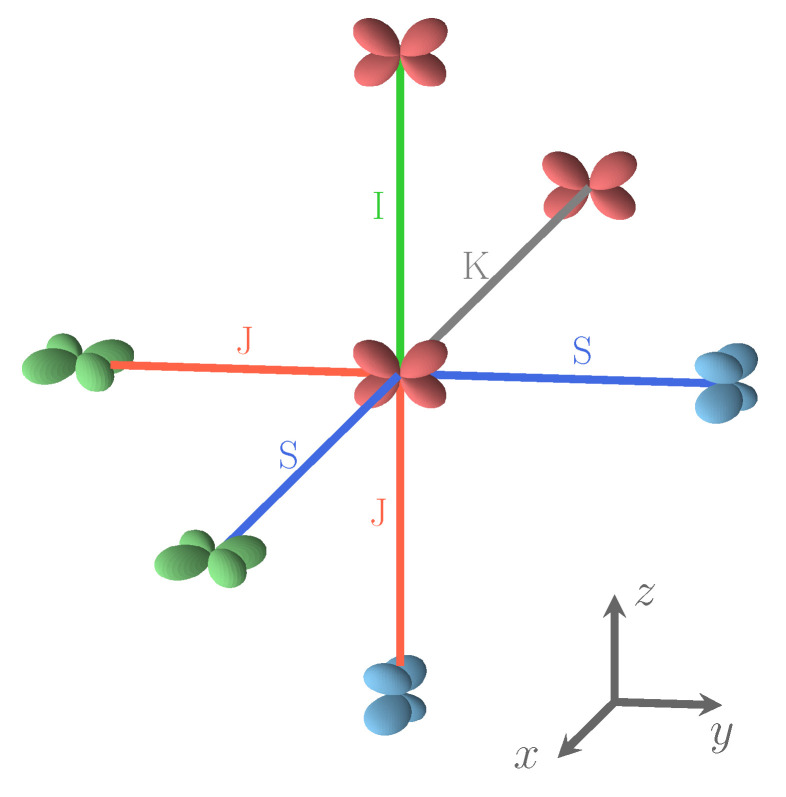
The four relative orientations (and then interactions) between first neighbor Fe atoms. Interaction types depend on *d* orbital labels and on the direction defined by the two Fe atoms. S and J interactions involve different *d* orbitals, and I and K connect identical *d* orbitals. In the Cartesian frame shows in the figure, d${}_{xy}$ is green, d${}_{xz}$ is blue, and d${}_{yz}$ is red. For details, see text.

The PBE0, B3LYP, and HSE06 populations are very similar, at 1.99 |e|, and the UHF one is only slightly higher at 2.00 |e|. In the various S222 configurations, the doubly occupied orbital can also be $d_{xz}$ or $d_{yz}$; the second columns show that the maximum deviation from the REF value is as small as 0.001 |e|. The same is true for the other four *d* orbitals, whose population is not shown, and is close to one. The lowest part of the table reports the non-null components of the quadrupole tensor (evaluated through a Mulliken partition of the charge density) of the atoms of the unit cell. It should be underlined that the dipole moments on all atoms are null for symmetry reasons. The quadrupole of the Fe atom is by far larger than the ones of the F and K atoms. The five α*d* electrons on Fe generate a (nearly) spherical charge distribution (and then null quadrupole components in the spherical harmonic basis here used), that is broken by the β electron. The only component of the quadrupole on Fe is then xy, xz, or yz according to the double occupancy on the various Fe atoms, as illustrated by Figure 2.

**Figure 3:**
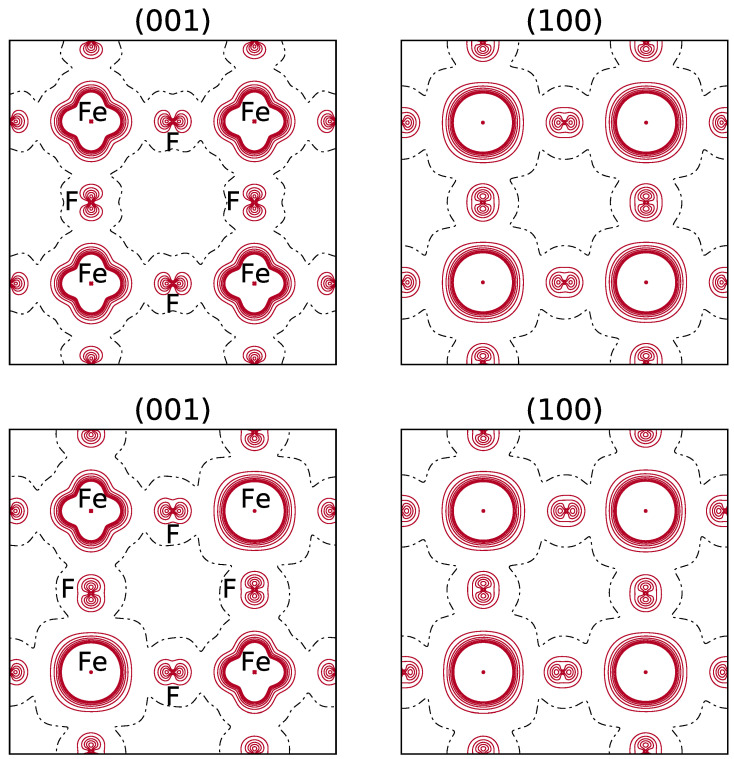
**Top**: B3LYP spin density maps of the primitive cell in the xy (**left**) and xz (**right**) planes. FM solution. The $d_{xy}$ orbital is doubly occupied. **Bottom**: the spin density function for the most stable configuration of the (044) composition (see the top left panel in Figure 1 for the occupancy of the d orbitals of the 8 Fe atoms). The separation between contiguous isodensity lines is 0.01 |e|a${}_{0}^{-3}$; the function is truncated in the core region at ±0.08 |e|a${}_{0}^{-3}$. Continuous, dashed and dot-dashed lines correspond to positive, negative and null values of the spin density, respectively.

It should be noticed that in the spin density maps, see Figure 3, referring to the REF case (β electron in d${}_{xy}$), the excess of α electrons due to d${}_{xz}$ and d${}_{yz}$ appears, whereas for electostatics the doubly occupied orbital, d${}_{xy}$, is providing the dominant contribution.

**Figure 4:**
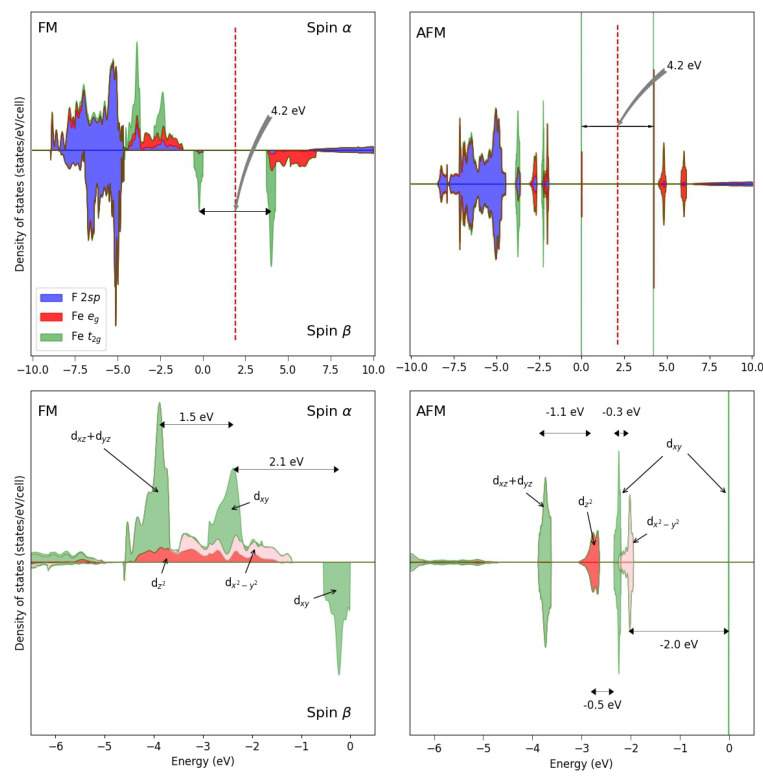
Density of states of the FM (left) solution of KFeF${}_{3}$ at the B3LYP level. For comparison, also the AFM DOS is shown to the right. The bottom panels are a zoom of the top panels in the highest valence energy region. The energy difference between the three main peaks (FM solution) is: 0.065 E${}_{h}$ between $d_{xz,yz}^{\alpha }$ and $d_{xy}^{\alpha }$, and 0.085 E${}_{h}$ between the α and β of the latter.

The Jahn–Teller split in KFeF${}_{3}$ is clearly evident from the DOS (see Figure 4, with the FM and AFM solutions), showing that:

(a) The *d* states occupy the top of the valence band.
(b) The $d_{xz}$ and $d_{yz}$ levels are the most stable at about 3.6 eV from the top of the valence band (see the left panel corresponding to the FM solution); their band is only 35 mE${}_{h}$ (0.94 eV) large.
(c) The $d_{xy}$ band is no more degenerate with the two other $t_{2g}$ levels; it is less stable than the other *d* levels; its α component is higher by about 66 mE${}_{h}$ than the $d_{xz}$ and $d_{yz}$ peaks; the β peak is 70–110 mE${}_{h}$ higher than the α ones.

The energy difference between the cubic structure in which the β electron on Fe is uniformly distributed on the three $t_{2g}$ orbitals and the tetragonal one in which a single $t_{2g}$ orbital is doubly occupied can be decomposed in a Jahn–Teller contribution (or electronic relaxation) evaluated at the cubic geometry reducing the symmetry from cubic ($Pm\bar{3}m$) to tetragonal ($P\frac{4}{m}bm$) and shifting one of the $t_{2g}$ (see the EIGSHIFT option in the CRYSTAL manual), and then imposing the (1,0,0) instead of the (1/3,1/3,1/3) occupancy of the three $t_{2g}$ orbitals, and the change in energy from the metrically cubic tetragonal solution and its geometrically optimized analog. It turns out that the Jahn–Teller energy contribution is three orders of magnitude larger than the geometry contribution: 1.1 E${}_{h}$ compared to just 0.95 mE${}_{h}$ (or 25 meV). As a consequence of relaxation, the *c* lattice parameter becomes shorter than the *a* and *b* ones: 4.09 and 4.22 Å at the B3LYP level (PBE0 and HSE06 are very similar), and 4.12 Å versus 4.24 Å at the UHF level. The Fe–F distances are just half the lattice parameters, then 2.05 and 2.12 Å in B3LYP (two and four neighbors, respectively).

### 3.1. The Orientation of the Doubly Occupied *d* Orbital

The B3LYP energies, cell parameters, volumes, kind of *d*–*d* interaction for the full set of S222 configurations are shown in Table A3, Table A4 and Table A5.

**Figure 5:**
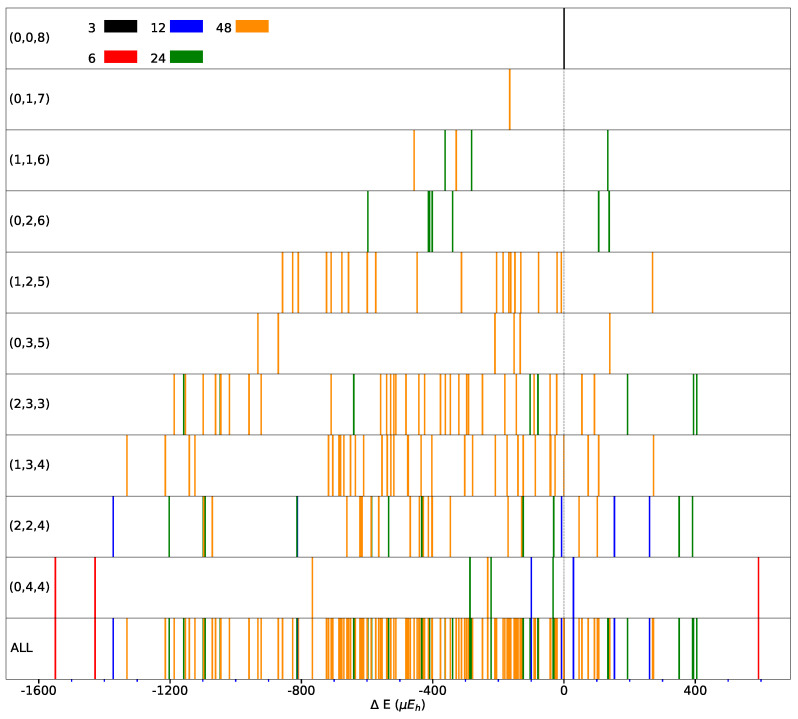
Distribution of the SICs as a function of the energy difference with respect to the REF case (indicated by the vertical dashed line) for CECs (Classes of Equivalent Composition) accessible using the S222 supercell. CEC’ representative *composition* is indicated in each panel (left). Panel “ALL” shows all the 162 SICs resulting from the 10 CECs. Different colors indicate different multiplicities. B3LYP energy in E${}_{h}$ per two f.u. (For interpretation of the reference to colors in this figure legend, the reader is referred to the web version of this article.)

The spanned energy range for the FM solutions is illustrated in Figure 5, where the multiplicity and then the symmetry of the SICs is also provided (see top left of the figure, and colors).

It should be noticed that the low symmetry SICs (largest multiplicity) cluster in the central part of the energy interval and that the most and less stable configurations are characterized by high symmetry. The central part of the energy interval is densely populated, while the edges are less populated. This is a consequence of the *relatively small* size of the supercell.

Only 21 of the 162 SICs are less stable than REF. At $n^{xy}$ constant, the most stable configuration of ($0,n^{xz}\neq 0,n^{xy}$) is always more stable than any of the ($n^{yz}\neq 0,n^{xz}\neq 0,n^{xy}$). This suggests that a few relative orientations of the *d* orbitals are stabilizing.

Figure 2 illustrates the four possible relative orientations of the d orbitals (and then quadrupoles) on first neighbors Fe atoms. Interestingly, the two most and the less stable configurations of the full set (see Figure 1, and labels 1, 2, and 162 in Table A3) belong to (0,4,4).

Looking in Figure 1 at the relative orientations of the doubly occupied *d* orbitals along the Cartesian axis a (or *x*), b (or *y*) and c (or *z*) between two first neighbor iron atoms, the stablest configurations are characterized by only two (out of four) types of relative orientations, the most unstable by three.

**Figure A1:**
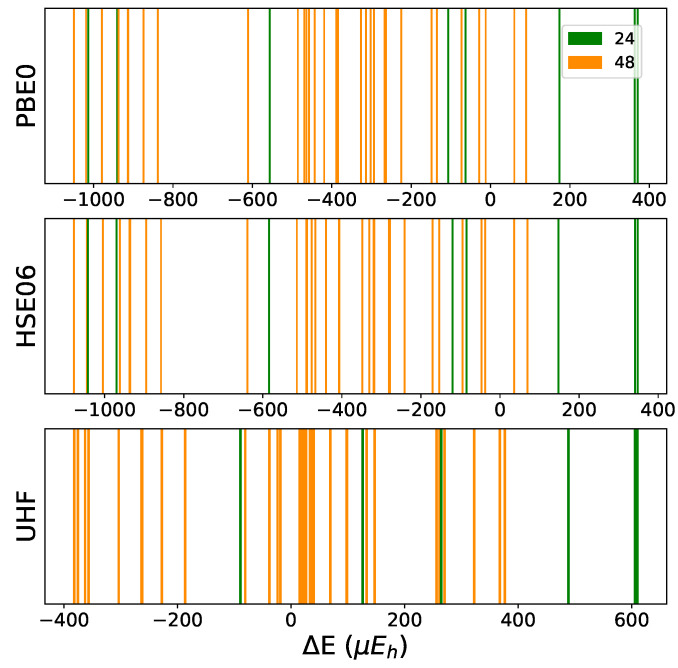
Distribution of the SICs belonging to the (2,3,3) CEC in relative total energy with respect to REF or (0,0,8) for 3 functionals: PBE0, HSE06 and UHF. Energy in $\mu E_{h}$ per 2 f.u. For each SIC, the multiplicity is indicated. The B3LYP case is in Figure 5 ((2,3,3) panel) in the main text.

**Figure A2:**
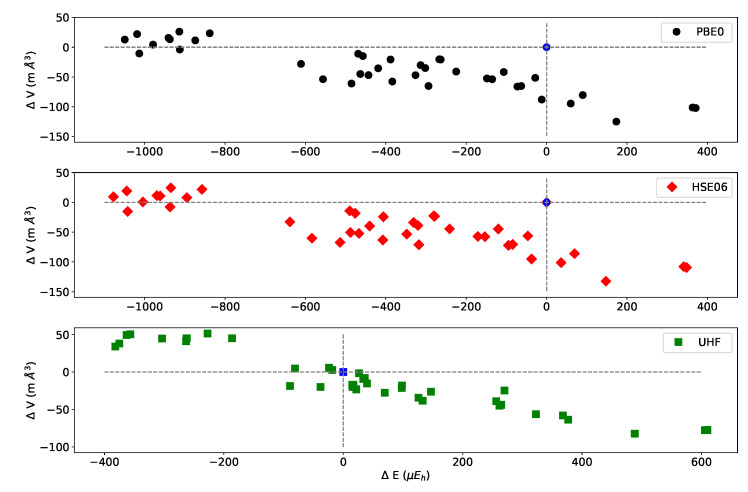
Relative volume versus relative total energy with respect to REF (blue symbol) of the symmetry independent classes (SIC) belonging to the (2,3,3) CEC, for 3 functionals: PBE0, HSE06, and UHF. Volume and energy per 2 f.u.

In order to test the influence of the Hamiltonian on the presented results, calculations have been performed on the (2,3,3) SICs using PBE0, HSE06, and UHF. The (2,3,3) composition has been chosen because it corresponds to the largest number of configurations (1680) and SICs (39). The results are shown in Figure A1 and Figure A2, and Table A6, Table A7 and Table A8. There are many interesting points to note:

(a) The energy difference with respect to REF spans from −1187 to +406 μE${}_{h}$ for B3LYP, from −1050 to +358 μE${}_{h}$ for PBE0 and from −1077 to +348 μE${}_{h}$ for HSE06 per two f.u.. Then not only the maximum and minimum energies, but also the spanned interval is about the same in the three cases.
(b) In the B3LYP, PBE0, and HSE06 cases, 34 out of 39 SIC (corresponding to 1512 out of 1680 configurations) are more stable than the fully ordered one, shown in the first row of the tables.
(c) Additionally, the volumes obtained with PBE0, HSE06 and UHF are very similar to the B3LYP ones.
(d) In the UHF case, trends are very similar to the one obtained for hybrids; ΔE values, however, are smaller by about a factor 3, due to the larger localization of the d electrons.

The above numbers provide a clear evidence that the fully ordered case is less stable than many “mixed” configurations, but not of all of them. From data in Table A5, containing the optimized lattice parameters (obtained at B3LYP level) of the S222 cells for each SIC, the cell distortion from cubic metric, δ= max(a,b,c)-min(a,b,c), strongly depends on the *composition*. The REF cell shows the maximum distortion (δ=0.24 Å) that reduces rapidly as the *composition* allows even patterns: δ<0.10 Å in (1,3,4), (2,2,4), and (2,3,3) configurations (more than two-thirds of the full set) and δ≈0.03 Å in the (2,3,3) patterns. The cell appears, however, as cubic, after averaging over the SICs and the possible orientations of the configurations (for example 0,4,4), (4,0,4), and (4,4,0)).

We can now try to identify the origin of the relative stability of many “mixed” configurations with respect to the fully ordered one. In principle, there are three variables that can stabilize one configuration with respect to another:

(a) A different charge distribution. We already commented that the net charges and the quadrupole on Fe are extremely similar in all configurations; so we can conclude that this variable has essentially no effect on energy differences. This statement obviously does not include the different orientations of the quadrupole on the Fe ions.
(b) A different geometry. We already commented that the cell volume of the configurations shows extremely small variations suggesting very similar geometries. The lattice parameters of the most stable and the less stable SICs are very close, as Table A5 shows. The small volume variations hide however dissimilar local situations around iron atoms. A tetragonally deformed octahedron with two apical (2.047 Å) and four equatorial (2.108 Å) distances characterizes REF. The difference between the Fe–F distances is even larger in the most stable configuration (2.122/2.109/2.032 Å); such a large difference is, however, not maintained in all configurations, for example, it reduces to 0.03 Å in the less stable configuration (Figure 1). It is not easy, however, to correlate many small distance differences to energy variations. However, certainly many small differences in the Fe–F and Fe–Fe distances can contribute with a non-negligible percentage to the energy stabilization/destabilization.
(c) The different quadrupolar interaction in the various configurations. As already anticipated, the quadrupoles on the Fe atoms is expected to play the most important role in the stabilization of some configurations with respect to others. This effect is, however, not easily evaluated, because, as discussed at point b, when the occupancy of the $t_{2g}$*d* orbital is changing, also the Fe–F distances are changing. So there is certainly a coupled geometry-electrostatics effect in the stabilization.

**Figure 6:**
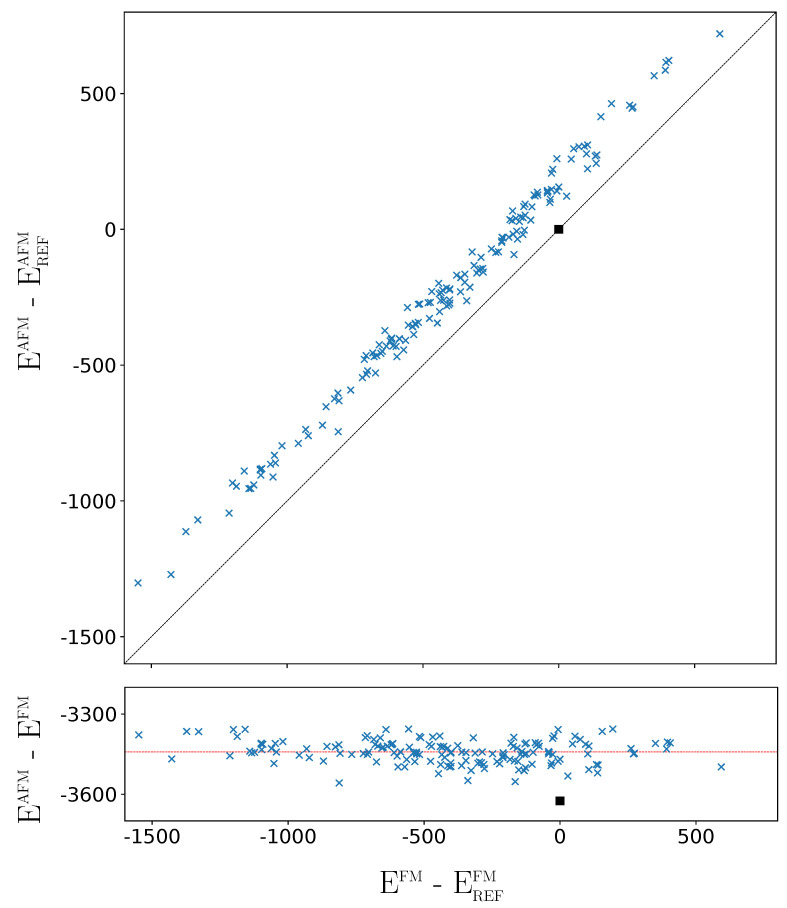
Difference between the AFM energy of the 162 SICs with respect to the REF case (top panel), and difference between the AFM and FM energies (bottom panel) as a function of the relative FM energy. The horizontal line corresponds to the average stabilization energy of the AFM solutions with respect to the FM ones. In both panels, energy at the B3LYP level (for 2 f.u. in μE${}_{h}$). The square indicates the REF case.

The above analysis refers to the FM solutions. How different is the situation when the G-AFM solutions of the 162 SICs are considered? The G-AFM fully ordered case (REF) is stabilized by 3625 μE${}_{h}$ per 2 f.u. with respect to the REF FM solution (see the square in the bottom panel of Figure 6, showing that the REF stabilization is the largest one).

The FM-AFM energy difference is more than twice the energy difference between the most stable and the REF FM solutions (1549 μE${}_{h}$, see caption of Figure 1, and Table A3). However the FM-AFM energy differences of the 162 SICs are very similar, with a mean value of −3443 μE${}_{h}$ and a standard deviation of 45 μE${}_{h}$.The orbital disorder is slightly less efficient for the AFM than for the FM solutions, by about 180 μE${}_{h}$, a small fraction of the energy range associated to disorder. As shown by the top panel of Figure 6, the disorder stabilisation effects for FM and AFM are strongly and linearly related, and the stability order of the 162 SICs is not significantly modified. For example, the relative stability of five most stable and the 10 least stable configurations is the same for FM and AFM. In the densely populated central energy interval, where SICs differ by few μE${}_{h}$, the stability order of the 162 AFM SICs is slightly different from the FM one. Note, in particular, that the REF configuration, that in the FM list (see Table A3) is number 141, in the AFM list (Table A4) is number 113, due to its large stabilization shown in Figure 6, and becomes more stable than the *columnar* [14] (see below) configuration, that is now at number 123.

In summary, the small variations between the FM and AFM cases, on the one hand, do not alter the analysis performed in this section on the basis of the FM solutions; on the other hand, confirm that the energy differences among the 162 SICs are mostly dictated by electrostatics, and are nearly independent from the magnetic interactions determining the FM-AFM energy differences. The maximum FM-AFM energy difference is observed for REF, that realizes the maximum Pauli pression of the majority spin electrons of F due to the *d* electrons of Fe; it is minimum for the most stable configurations that, with a more effective packing, also minimize the Pauli pression on F.

**Figure 7:**
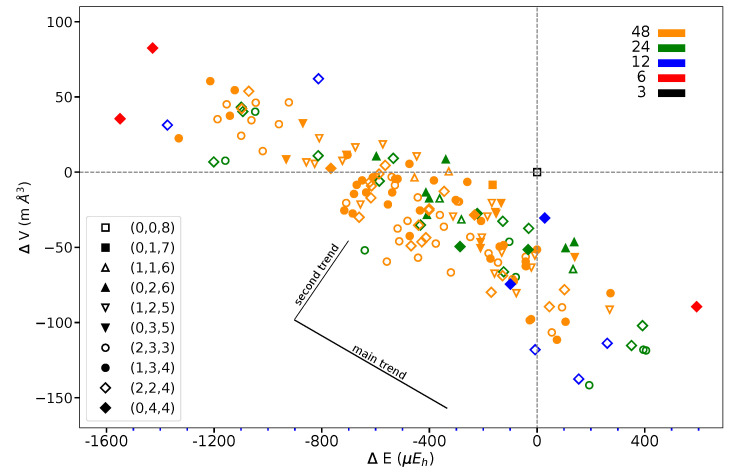
Volume difference *versus* energy difference with respect to REF or (0,0,8) as obtained for the 162 SICs of the S222 supercell of KFeF${}_{3}$. The figure legend associates symbols and CECs, color, and multiplicity, as in Figure 5. The two trends described in the text are sketched. Energy in E${}_{h}$ and volume in Å${}^{3}$ refer to two f.u.

Figure 7 shows that the volume variation along the full series of 162 SICs does not exceed 0.1% of the REF volume (140.348 Å${}^{3}$ per 2 f.u.).

This suggests that the structural changes are very small. The volume/energy figure defines a broad band. At first, considering lowest and highest energy points, volume and energy seem to be anti-correlated. However, looking at SICs belonging to a given composition, a second trend appears, orthogonal to the first one, as shown in the figure.

**Figure A3:**
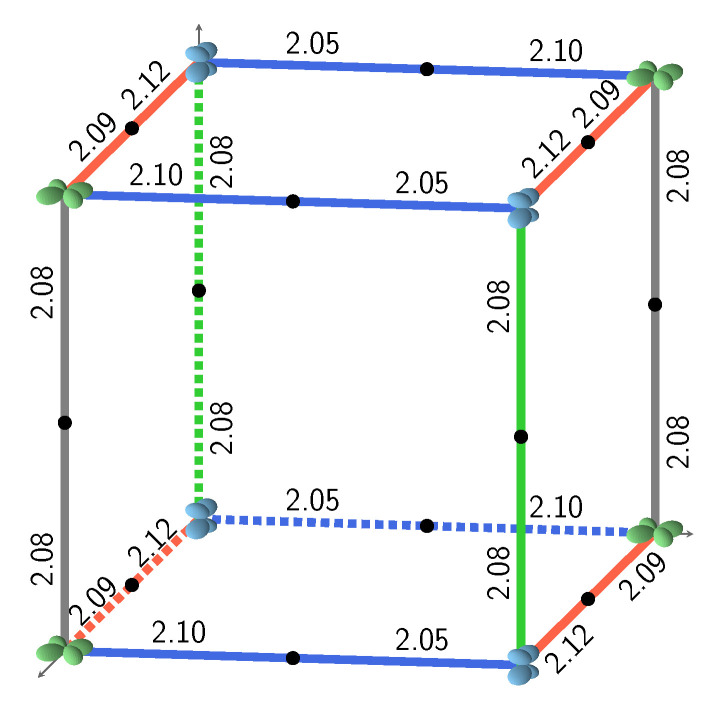
The configuration indicated as *columnar* by Varignon et al., see Table III of Reference [14] of the main text.

In a recent paper Varignon et al. [14] investigated at the FM level three configurations of a KFeF${}_{3}$ supercell containing 4 Fe atoms that they call *single*, *columnar*, and *3D checkerboard*. The first one coincides with the REF configuration, and the third one with the most stable configuration shown in Figure 1. The *columnar* configuration is shown in Figure A3; it has the same basal plane as the most stable one of Figure 1; all planes along the *c* lattice parameter are the same as the basal plane. This configuration is not the second-most stable one; actually it is at number 124 in the list of the 162 FM SICs shown in Table A4. A quantitative comparison with the present results is not easy, because, (i) in Varignon et al. the geometry is not fully optimized; (ii) They use a plane-wave basis set, with pseudopotentials, whereas here we use an *all electron* Gaussian type basis set. (iii) The Hamiltonian in their case is LDA+U. We are, then, unable to establish a quantitative correspondence with the B3LYP, PBE0, HSE06, and UHF functionals used here. Their *3D checkerboard* and *columnar* energies (see their Table III) are 1984 and 1286 $\mu E_{h}$ per 2 f.u. lower than *single*; the present numbers are 1549 and 99, 1359 and 83, and 1390 and 98 $\mu E_{h}$ for B3LYP, PBE0, and HSE06, respectively.

### 3.2. Simple Models for Describing the Stability Order

We can look for a simple model for describing the energy of the various configurations. We suppose that the interactions are additive and that the interaction energy of each Fe with the other Fe atoms is the sum of the individual interactions with the first Fe neighbors, and that farther neighbors are irrelevant. Variation of the Fe–Fe and Fe–F distances within a given configuration, and for the various configurations is not taken into account.

Four types of interactions are rationalized on the basis of the relative orientation of the *d* shells on Fe, as shown in Figure 2. If, on the two Fe atoms, unlike *d* orbitals are doubly occupied, the interaction is J, if the atoms are connected along the direction (x, y, or z) appearing in both *d* orbitals (i.e., $d_{xz}$-$d_{yz}$ along *z*); in the other cases the interaction is S (i.e., $d_{xz}$-$d_{yz}$ along *x* or *y*). If the doubly occupied orbital on the two Fe atoms is the same, the interaction is I, if the atoms are aligned along one of the two directions appearing in the *d* orbitals (i.e., $d_{xy}$-$d_{xy}$ along *x* or *y*); it is K in the other cases ($d_{xy}$-$d_{xy}$ along *z*).

Looking at the Fe–F–Fe path, we observe that I, J and K are symmetric with respect to the two Fe atoms, so that F is expected to be midway. The S interaction, on the contrary, is not symmetric, and F is expected to be closer to one of the Fe atoms. For example, if one of the *d* orbitals is, say xz (on Fe${}_{1}$), and the other xy (on Fe${}_{2}$), and the direction of the bond is *x*, then F will be closer to Fe${}_{2}$. This local point of view must, however, take into account the various constraints deriving from the 3D structure. The maximum difference between the shortest and the longest Fe–F bond length, in the set of the 162 SICs, is observed for S (0.14 Å); for the other three interactions it is ≈0.06 Å. The mean short and long bond for I, J, and K interactions are very close, 2.086/2.098, 2.087/2.098 and 2.075/2.088 Å, respectively, but differ significantly for S: 2.049/2.122 Å.

S222 (with 8 f.u.) is characterized by 24 interactions; however, for symmetry reasons, only 12 are shown in Figure 1. The four configurations shown in the figure support the symmetry statement concerning the F position. The same figure shows that the three most stable configurations are characterized by a large number of S interactions (16 out of 24); in the less stable configuration, on the contrary, there is no S interaction.

What about I and J? Consider the two most stable configurations, both belonging to the (0,4,4) composition. They both are characterized by 16 S interactions. The most stable, however, has 8 J, whereas in the other there are 8 I. Then exchanging J with I destabilizes the configuration. Looking now at Figure 7, where the two energies appear to the left, top (red diamonds), we observe that the same exchange increases the volume (the second trend indicated in the figure).

Such a simple model, based on the S, I, J, and K interactions, is unable to reproduce the computed energies. Its major deficiency is its failure to approach the zero energy of the reference configuration.

**Figure A4:**
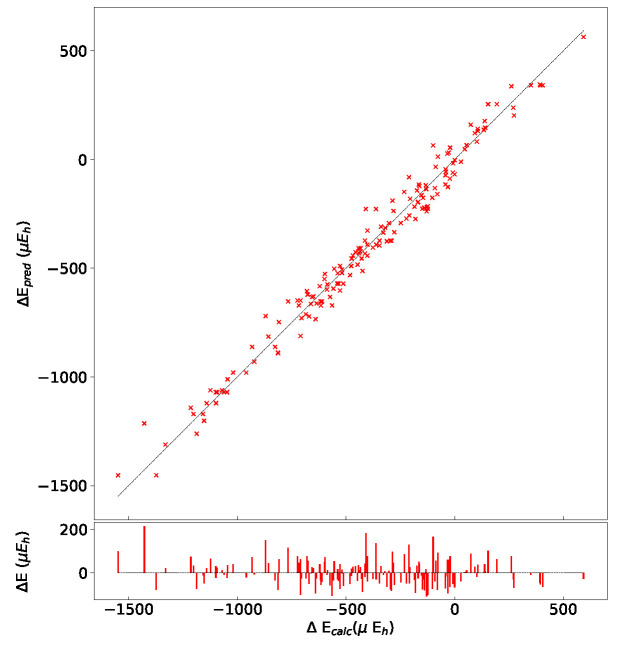
Comparison between the B3LYP relative energies ($\Delta E_{\mathrm{calc}}$) and the relative energies predicted according to the 12 interaction model described in the text ($\Delta E_{\mathrm{pred}}$) for the 162 SICs. Energies refer to 2 f.u. Top: E${}_{\mathrm{pred}}$ as a function of E${}_{\mathrm{calc}}$. The even distribution with respect to the perfect agreement straight line (dashed line) confirms the good quality of the model. Bottom: Deviation ($\Delta E_{\mathrm{pred}}$-$\Delta E_{\mathrm{calc}}$) as a function of $\Delta E_{\mathrm{calc}}$. The two panels share the horizontal scale. The energy differences are a small fraction of the energy interval covered by the set of SICs; only 4 exceed 10% of the energy interval.

A more sophisticated model has then been explored, taking simultaneously into account the three interactions associated to each Fe site. A three-letter string (i.e., ISS or IJK) is associated to each site, where the letters refer to the four Fe–Fe interactions previously defined. Such model keeps partial track of the connectivity between the Fe–Fe couples sharing a common Fe. Starting from the cubic symmetry of the aristotype structure of perovskite, and in order to keep the number of parameters as small as possible, anagrams are merged in a unique parameter (this means that we consider equivalent, for example, IJK, IKJ, JIK, JKI, KIJ, and KJI). The model contains 12 parameters. Its ability to mimic the dataset is significantly improved with respect to the model based simply on the four interactions S, I, J, and K. It produces 143 independent energies for the 162 SICs. The model is as follows:

(1) $$\begin{array}{cc} E^{i}=\; & n_{\mathrm{SSS}}^{i}C_{\mathrm{SSS}}+n_{\mathrm{JSS}}^{i}C_{\mathrm{JSS}}+n_{\mathrm{ISS}}^{i}C_{\mathrm{ISS}}+n_{\mathrm{IIS}}^{i}C_{\mathrm{IIS}}+n_{\mathrm{JJS}}^{i}C_{\mathrm{JJS}}+n_{\mathrm{IIK}}^{i}C_{\mathrm{IIK}}+n_{\mathrm{IJS}}^{i}C_{\mathrm{IJS}}+n_{\mathrm{JKS}}^{i}C_{\mathrm{JKS}}\\ \; & +n_{\mathrm{IKS}}^{i}C_{\mathrm{IKS}}+n_{\mathrm{IJK}}^{i}C_{\mathrm{IJK}}+n_{\mathrm{KSS}}^{i}C_{\mathrm{KSS}}+n_{\mathrm{JJK}}^{i}C_{\mathrm{JJK}} \end{array}$$

where $n_{\alpha \beta \gamma }^{i}$ and $C_{\alpha \beta \gamma }$ are the number of Fe sites involved in simultaneously α, β, and γ interactions and the corresponding effective coefficients of interaction (ECI), respectively. Obviously, for S222, $\sum n_{\alpha \beta \gamma }^{i}=8$. The AFM ECIs as resulting from the best fit are: −744.8, −595.6, −523.2, −26.4, +110.0, −0.0, +121.6, +142.0, +97.2, +352.8, +355.2, and +489.6 μE${}_{h}$. The RMSE (61.4 μE${}_{h}$ per 2 f.u.), and the MAE (214.4 μE${}_{h}$ per 2 f.u.) represent 3% and 10% of the energy interval covered by the SICs, respectively, and suggest that the model provides a very satisfactory representation of the data. The FM ECIs are very similar: −824.8, −726.0, −606.8, −64.8, −66.4, −0.8, +17.2, +47.6, +55.6, +282.0, +304.0, and +335.2 μE${}_{h}$, with three large and negative and three large and positive ECIs. The quality of the fit is documented by Figure A4. The energy of the (0,0,8) REF configuration is very well reproduced: it is characterized by 8 IIK interactions whose ECI is 0.0 and −0.8 μE${}_{h}$ for the AFM and the FM cases, respectively. It is interesting to note that the stabilizing character of these three fold interactions decreases as their “S content” decreases, the exception being KSS (+355.2 μE${}_{h}$).

These ECI call for some comments:

(i) Only configurations containing two or three different $t_{2g}$*d* orbitals doubly occupied can be more stable than REF characterized by a single type of doubly occupied *d* orbital;
(ii) It is impossible to build a configuration containing only S interactions, so the stablest configuration found in this work is the stablest for any supercell within the present approximations;
(iii) The high stability of the configurations with a large number of S interactions can be related to the orientation of the quadrupole on Fe atoms; the S arrangement corresponds to the herringbone structure reported for molecular crystals characterized by a dominant contribution from the molecular quadrupole, such as benzene [41];
(iv) The flexibility of the Fe–F bonds allowed by the local asymmetry of the S interaction is expected to contribute to the stability of the configurations.

## 4. Conclusions and Perspectives

Many transition metal compounds of the first row, with the 3*d* shell partially occupied, undergo a Jahn–Teller deformation, when the number of degenerate orbitals in a given sub-shell is larger than the number of α or β electrons to be allocated.

In the perovskite compounds KMF${}_{3}$, this is the case of 6 (Sc, $d^{1}$; Ti, $d^{2}$; Cr, $d^{4}$; Fe, $d^{6}$; Co, $d^{7}$; and Cu, $d^{9}$) out of 10 systems of the family starting from KScF${}_{3}$ and ending with KZnF${}_{3}$. The cubic symmetry of the ideal perovskite structure, with two *d* sub-shells containing three $t_{2g}$ and two $e_{g}$ orbitals, reduces to tetragonal as a consequence of the split of (say) the $d_{xy}$ from the $d_{xz}$ and $d_{yz}$ (Sc, Ti, Fe, Co), or of the $d_{z^{2}}$ from the $d_{x^{2}-y^{2}}$ orbital (Cr, Cu). The deformation of the unit cell in the cases of the Fe compound, explored here, is of the order of 0.13 Å (the lattice parameter c is shorter than a and b); the energy difference due to this tetragonal relaxation is as small as 1 m$E_{h}$.

There is, however, no reason for having the double occupancy in $d_{xy}$ rather than in $d_{xz}$ or $d_{yz}$; it is shown here that if a supercell (2 × 2 × 2) is built in which the three $t_{2g}$ orbitals are occupied on contiguous sites, in most of the cases an energy is reached which is lower than the one of the fully ordered case (all cells in the infinite lattice with the $d_{xy}$ orbital doubly occupied).

In particular, it is shown here that:

(a) The energy gain, for the most stable configurations, is of the order of 1550 μE${}_{h}$ per 2 Fe atoms when the hybrid B3LYP functional is used.
(b) PBE0 and HSE06 provide very similar results, quantitatively.
(c) It is shown that four relative orientations are possible between two Fe ions, each one containing one doubly occupied *d* orbital; the energy of the various configurations can be fully explained on the basis of this orientation, that reflects the relative interaction of the quadrupole of the Fe ions.
(d) A simple model is proposed, containing 12 parameters, that when fitted to the energy of the 162 SICs, provides values of the parameters that clearly explain the role of the various *d*–*d* interactions. The root mean square and the maximum absolute errors of the fitting are as small as 61 and 214 μE${}_{h}$.
(e) The small volume reduction when going from the most stable to the least stable configuration is also explained.
(f) The present results have been obtained by considering all possible configurations (6561) of all possible *compositions* (from (0,0,8) to (2,3,3)) in a cell containing 8 formula units (40 atoms, S222), represented by 162 SICs.

It should be underlined that the present calculations require the preparation of a large number of input files including the definition of the initial guess for the 8 Fe ions. All these manipulations are, however, performed automatically through scripts available to the reader. The present calculations exploiting the symmetry both to catalog the *d* patterns and to simplify the *ab initio* calculations, performed with an *all electron* scheme and (full range) hybrid functionals, are relatively cheap. The tools implemented for the present analysis are general, so that in the near future extensions are foreseen to the following sets of compounds:

(a) KScF${}_{3}$, KTiF${}_{3}$, and KCoF${}_{3}$, that are, respectively, $d^{1}$, $d^{2}$, and $d^{7}$, to confirm the present general findings on KFeF${}_{3}$.
(b) KCrF${}_{3}$ and KCuF${}_{3}$, for which the Jahn–Teller effect is much larger both in geometry and energy.
(c) The corresponding oxide compounds with $d^{1}$,$d^{2}$, $d^{6}$ and $d^{7}$ configuration.
(d) In a longer term, compounds of the lanthanide family, for which the scenario of the Jahn–Teller split of the seven *f* orbitals has received a limited attention.

## Figures and Tables

**Table 1:** Atomic net charges (C) and population of the doubly occupied *d* orbital (q), evaluated according to a Mulliken analysis. For each considered functional, the first column (REF) gives the net charges computed for the primitive FM cell, and the second column (SOL) the maximum absolute difference observed for all positions (eight for Fe, for example) and for all possible configurations. Numbers are in electrons. The $d_{xy}$ orbital is doubly occupied in the primitive cell; in the various configurations $d_{xz}$ and $d_{yz}$ can be doubly occupied, alternatively. In the bottom five lines the non-null elements of the atomic quadrupole Q tensor of the primitive cell (again computed according to a Mulliken partition of the charge) are reported. F${}_{a}$ and F${}_{e}$ indicate apical and equatorial F atoms. The quadrupoles are in Bohr${}^{2}$.

				PBE0		B3LYP		HSE06		UHF	
				REF	SOL	REF	SOL	REF	SOL	REF	SOL
*C*	{	Fe		+1.725	0.012	+1.712	0.012	+1.723	0.012	+1.852	0.008
		F${}_{a}$		−0.871	0.004	−0.863	0.005	−0.870	0.005	−0.931	0.004
		F${}_{e}$		−0.890	0.000	−0.885	0.000	−0.889	0.001	−0.943	0.000
		K		+0.926	0.000	+0.920	0.000	+0.926	0.000	+0.966	0.000
q		Fe	xy	1.992	0.000	1.992	0.001	1.992	0.000	1.997	0.001
*Q*	{	Fe	xy	−0.4082		−0.4068		−0.4095		−0.4263	
		F${}_{a}$	z${}^{2}$	−0.0156		−0.0081		−0.0050		−0.0629	
		F${}_{e}$	xy	−0.0032		−0.0092		−0.0101		+0.0245	
		F${}_{e}$	$x^{2}-y^{2}$	+0.0078		+0.0372		+0.0473		−0.1247	
		K	xy	+0.0055		+0.0061		+0.0055		+0.0013	

**Figure A5:**
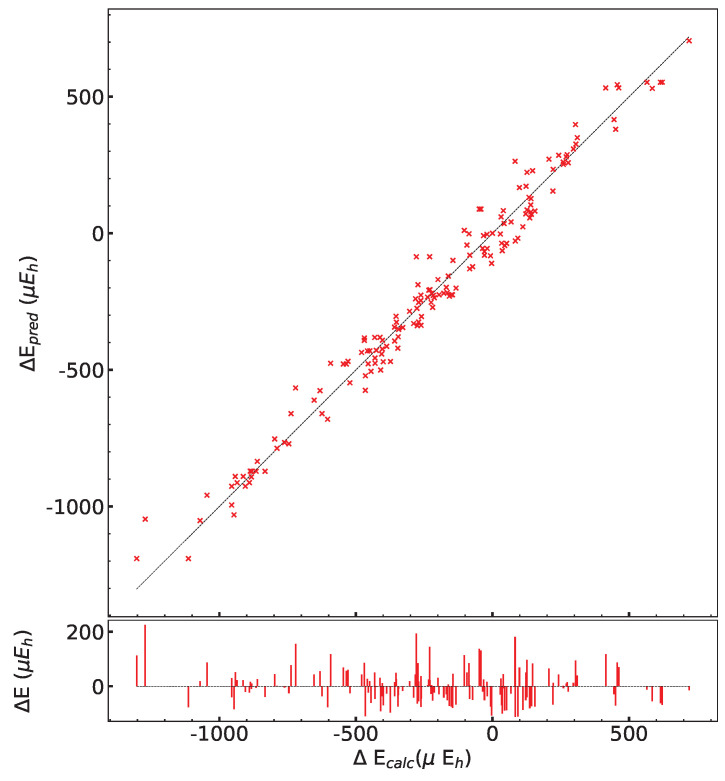
As in previous Figure A4, for the AFM solutions.

## Data Availability

The data that supports in this study are available within the article, its supplementary material and openly available in NOMAD repository, reference number https://doi.org/10.17172/NOMAD/2022.06.03-2, accessed on 10 February 2023.

## Abbreviations

The following abbreviations are used in this manuscript:

FM	Ferromagnetic
AFM	Anti-ferromagnetic
TM	Transition metal
REF	Reference case, or fully ordered case
S222	Supercell 2 × 2 × 2
SIC	Symmetry independent class
CEC	Class of Equivalent Composition

## Appendix A

**Table A1:** Fe *all electron* basis set used in the present calculations.

26	7					
		0	0	8	2.0	1.0
				316,081.00000	0.00023	
				44,436.07169	0.00198	
				9407.88758	0.01133	
				2482.45939	0.05006	
				759.29764	0.16681	
				265.48720	0.36200	
				103.70697	0.40461	
				42.68987	0.14310	
		0	1	6	8.0	1.0
				908.50077	−0.00522	0.00697
				219.24222	−0.05903	0.05026
				72.29301	−0.14733	0.19273
				27.61710	0.20704	0.40918
				11.49074	0.72716	0.41250
				4.67579	0.30002	0.13239
		0	1	4	8.0	1.0
				46.29660	0.01245	−0.02242
				15.70488	−0.27343	−0.08216
				6.77928	−0.74723	0.22377
				2.87979	1.04415	1.19671
		0	1	1	0.0	1.0
				1.18217	1.00000	1.00000
		0	1	1	0.0	1.0
				0.45910	1.00000	1.00000
		0	3	4	6.0	1.0
				38.41816	0.03961	
				10.70357	0.20699	
				3.62270	0.50426	
				1.24468	0.64708	
		0	3	1	0.0	1.0
				0.39118	1.00000	

**Table A2:** K and F *all electron* basis set used in the present calculations.

19	5					
		0	0	8	2.0	1.0
				170,602.10967	0.00022	
				23,423.76869	0.00200	
				4917.11428	0.01146	
				1304.66894	0.04981	
				404.20606	0.16262	
				142.59971	0.35279	
				55.44223	0.40663	
				22.29157	0.14591	
		0	1	6	8.0	1.0
				468.62462	−0.00467	0.00633
				112.32579	−0.05677	0.04549
				36.54740	−0.14030	0.17492
				13.64525	0.21375	0.37901
				5.44569	0.71759	0.41735
				2.02428	0.29729	0.16206
		0	1	5	8.0	1.0
				19.50859	−0.00354	−0.02340
				6.13810	−0.28502	−0.08654
				2.79749	−0.61996	0.22695
				1.14560	1.04144	1.31866
				0.42945	1.28719	1.29059
		0	1	1	0.0	1.0
				0.51624	1.00000	1.00000
		0	1	1	0.0	1.0
				0.16791	1.00000	1.00000
9	4					
		0	0	7	2.0	1.0
				10,139.60067	0.00110	
				1517.42324	0.00850	
				348.08291	0.04183	
				100.56220	0.14604	
				33.74770	0.33609	
				12.83710	0.40569	
				5.27944	0.16871	
		0	1	3	8.0	1.0
				39.45194	−0.06901	0.07752
				9.17932	−0.39853	0.45487
				2.75061	0.48002	1.30646
		0	1	1	0.0	1.0
				0.86820	1.00000	1.00000
		0	1	1	0.0	1.0
				0.24838	1.00000	1.00000

**Table A3:** B3LYP relative energy (ΔE in μE${}_{h}$ per 2 f.u.) and volume (ΔV in mÅ${}^{3}$ per 2 f.u.) for the FM solution with respect to REF (in bold, E${}_{REF}$ = −4326.636451 E${}_{h}$) of the 162 SICs labelled by increasing energy (L${}_{\mathrm{SIC}}$) and the number of the four first neighbor interactions as defined in the text (n${}_{S}$, n${}_{I}$, n${}_{J}$, and n${}_{K}$). The star in the first column indicates the configurations investigated by Varignon et al. (see Reference [14] in the main text), labelled (i), (ii), and (iii) in their Table III. They appear at L${}_{\mathrm{SIC}}$ = 1, 124 and 141.

L${}_{\mathrm{SIC}}$	n${}_{S}$	n${}_{I}$	n${}_{J}$	n${}_{K}$	ΔE	ΔV	L${}_{\mathrm{SIC}}$	n${}_{S}$	n${}_{I}$	n${}_{J}$	n${}_{K}$	ΔE	ΔV	L${}_{\mathrm{SIC}}$	n${}_{S}$	n${}_{I}$	n${}_{J}$	n${}_{K}$	ΔE	ΔV
**1 ***	16	0	8	0	−1549.3	35.5	55	12	0	10	2	−558.3	−59.5	109	8	2	10	4	−170.3	−79.5
2	16	8	0	0	−1428.3	82.5	56	12	4	6	2	−554.3	−20.5	110	8	8	4	4	−168.3	−20.5
3	16	0	8	0	−1373.3	31.5	57	12	6	4	2	−540.3	−3.5	111	4	12	2	6	−165.3	−8.5
4	16	0	8	0	−1331.3	19.5	58	12	6	4	2	−538.3	−15.5	112	8	4	8	4	−158.3	−67.5
5	16	6	2	0	−1214.3	59.5	59	12	8	2	2	−534.3	9.5	113	8	10	2	4	−152.3	−27.5
6	16	0	8	0	−1201.3	6.5	60	12	6	4	2	−528.3	−8.5	114	8	8	4	4	−155.3	−26.5
7	16	2	6	0	−1187.3	35.5	61	12	6	4	2	−527.3	−3.5	115	12	6	4	2	−145.3	−60.5
8	16	0	8	0	−1158.3	7.5	62	12	6	4	2	−517.3	−4.5	116	8	6	6	4	−140.3	−50.5
9	16	4	4	0	−1153.3	45.5	63	12	2	8	2	−518.3	−37.5	117	8	10	2	4	−133.3	−20.5
10	16	4	4	0	−1141.3	44.5	64	12	2	8	2	−512.3	−45.5	118	8	6	6	4	−131.3	−53.5
11	16	6	2	0	−1123.3	52.5	65	12	4	6	2	−481.3	−32.5	119	8	4	8	4	−128.3	−68.5
12	16	4	4	0	−1099.3	43.5	66	12	8	2	2	−474.3	3.5	120	12	10	0	2	−127.3	−32.5
13	16	2	6	0	−1099.3	24.5	67	12	4	6	2	−473.3	−43.5	121	8	6	6	4	−124.3	−50.5
14	16	4	4	0	−1097.3	42.5	68	12	2	8	2	−468.3	−48.5	122	8	4	8	4	−124.3	−66.5
15	16	4	4	0	−1093.3	40.5	69	8	8	4	4	−439.3	−4.5	123	12	8	2	2	−103.3	−46.5
16	16	6	2	0	−1071.3	53.5	70	12	10	0	2	−447.3	10.5	**124***	8	4	8	4	−99.3	−74.5
17	16	4	4	0	−1061.3	34.5	71	12	2	8	2	−442.3	−56.5	125	8	4	8	4	−91.3	−71.5
18	16	4	4	0	−1047.3	40.5	72	12	4	6	2	−440.3	−35.5	126	8	4	8	4	−87.3	−73.5
19	16	6	2	0	−1043.3	46.5	73	12	6	4	2	−435.3	−28.5	127	8	4	8	4	−79.3	−69.5
20	16	2	6	0	−1019.3	14.5	74	12	4	6	2	−433.3	−35.5	128	8	4	8	4	−77.3	−80.5
21	16	4	4	0	−959.3	31.5	75	12	4	6	2	−428.3	−46.5	129	8	6	6	4	−42.3	−59.5
22	12	4	6	2	−932.3	8.5	76	12	6	4	2	−443.3	−16.5	130	8	6	6	4	−42.3	−56.5
23	16	6	2	0	−922.3	46.5	77	8	8	4	4	−413.3	−13.5	131	8	6	6	4	−42.3	−64.5
24	12	8	2	2	−870.3	32.5	78	12	4	6	2	−413.3	−43.5	132	8	6	6	4	−40.3	−63.5
25	12	4	6	2	−857.3	6.5	79	8	8	4	4	−405.3	−19.5	133	8	8	4	4	−33.3	−51.5
26	12	4	6	2	−826.3	5.5	80	12	8	2	2	−383.3	−6.5	134	8	8	4	4	−31.3	−37.5
27	12	4	6	2	−813.3	10.5	81	12	6	4	2	−402.3	−25.5	135	8	2	10	4	−27.3	−99.5
28	16	8	0	0	−812.3	62.5	82	8	8	4	4	−401.3	−17.5	136	8	2	10	4	−22.3	−97.5
29	12	6	4	2	−809.3	22.5	83	12	6	4	2	−401.3	−24.5	137	8	6	6	4	−21.3	−63.5
30	12	6	4	2	−766.3	2.5	84	12	4	6	2	−376.3	−47.5	138	8	8	4	4	−8.3	−55.5
31	12	6	4	2	−723.3	7.5	85	8	8	4	4	−362.3	−17.5	139	8	0	12	4	−7.3	−118.5
32	12	2	8	2	−716.3	−25.5	86	12	6	4	2	−361.3	−28.5	140	8	8	4	4	−0.3	−51.5
33	12	6	4	2	−708.3	11.5	87	12	8	2	2	−345.3	−12.5	**141***	**0**	**16**	**0**	**8**	**0.0**	**0.0**
34	12	2	8	2	−709.3	−20.5	88	12	6	4	2	−346.3	−36.5	142	8	12	0	4	28.7	−30.5
35	12	6	4	2	−704.3	11.5	89	8	12	0	4	−339.3	7.5	143	8	4	8	4	45.7	−89.5
36	12	2	8	2	−685.3	−29.5	90	8	10	2	4	−327.3	0.5	144	8	2	10	4	54.7	−106.5
37	12	4	6	2	−680.3	−15.5	91	12	2	8	2	−320.3	−66.5	145	8	2	10	4	73.7	−111.5
38	12	8	2	2	−675.3	16.5	92	8	6	6	4	−312.3	−29.5	146	8	4	8	4	92.7	−89.5
39	12	4	6	2	−670.3	−11.5	93	12	8	2	2	−302.3	−22.5	147	8	6	6	4	101.7	−78.5
40	12	2	8	2	−661.3	−29.5	94	12	8	2	2	−296.3	−19.5	148	4	10	4	6	105.7	−51.5
41	12	4	6	2	−656.3	−21.5	95	12	8	2	2	−290.3	−19.5	149	8	4	8	4	105.7	−100.5
42	12	4	6	2	−650.3	−4.5	96	8	4	8	4	−286.3	−49.5	150	4	8	6	6	133.7	−63.5
43	12	0	10	2	−640.3	−51.5	97	8	8	4	4	−280.3	−30.5	151	4	10	4	6	137.7	−46.5
44	12	4	6	2	−635.3	−13.5	98	12	10	0	2	−278.3	−9.5	152	4	8	6	6	139.7	−56.5
45	12	4	6	2	−621.3	−6.5	99	12	6	4	2	−248.3	−43.5	153	8	0	12	4	154.7	−137.5
46	12	4	6	2	−617.3	−17.5	100	8	8	4	4	−232.3	−28.5	154	8	0	12	4	193.7	−141.5
47	12	4	6	2	−615.3	−9.5	101	8	8	4	4	−222.3	−27.5	155	8	4	8	4	260.7	−113.5
48	12	6	4	2	−610.3	−1.5	102	8	6	6	4	−210.3	−46.5	156	4	6	8	6	269.7	−91.5
49	12	6	4	2	−599.3	−3.5	103	8	6	6	4	−210.3	−50.5	157	4	6	8	6	272.7	−81.5
50	8	8	4	4	−597.3	10.5	104	8	6	6	4	−209.3	−32.5	158	4	4	10	6	350.7	−115.5
51	12	6	4	2	−587.3	−1.5	105	8	6	6	4	−205.3	−43.5	159	4	6	8	6	391.7	−102.5
52	12	6	4	2	−586.3	−5.5	106	8	8	4	4	−184.3	−29.5	160	4	4	10	6	394.7	−118.5
53	12	8	2	2	−573.3	18.5	107	8	4	8	4	−180.3	−54.5	161	4	4	10	6	404.7	−118.5
54	12	6	4	2	−564.3	4.5	108	8	4	8	4	−172.3	−58.5	162	0	8	8	8	592.7	−89.5

**Table A4:** B3LYP relative energy (ΔE in μE${}_{h}$ per 2 f.u.) and volume (ΔV in mÅ${}^{3}$ per 2 f.u.) for the AFM solution with respect to REF (in bold, E${}_{REF}$ = −4326.640076 E${}_{h}$, V${}_{REF}$ = 144.8500 Å${}^{3}$) of the 162 SICs labelled by increasing energy (L${}_{\mathrm{SIC}}$). For each SIC, the number of S, I, J and K interactions is indicated. The star in the first column indicates the configurations investigated by Varignon et al. (see Reference [14] in the main text), labelled i), ii), and iii) in their Table III. They appear at L${}_{\mathrm{SIC}}$ = 1, 123, and 113.

L${}_{\mathrm{SIC}}$	CEC	n${}_{S}$	n${}_{I}$	n${}_{J}$	n${}_{K}$	ΔE	ΔV	L${}_{\mathrm{SIC}}$	CEC	n${}_{S}$	n${}_{I}$	n${}_{J}$	n${}_{K}$	ΔE	ΔV
**1 ***	**(0,4,4)**	**16**	**0**	**8**	**0**	**−1302.8**	**99.1**	55	(2,2,3)	12	0	10	2	−373.5	20.1
2	(0,4,4)	16	8	0	0	−1271.5	110.1	56	(1,3,4)	12	6	4	2	−358.8	27.1
3	(2,2,4)	16	0	8	0	−1113.9	97.1	57	(2,2,3)	12	6	4	2	−358.3	38.1
4	(1,3,4)	16	0	8	0	−1070.6	86.1	58	(1,3,4)	12	4	6	2	−353.3	31.1
5	(1,3,4)	16	6	2	0	−1045.4	96.1	59	(1,3,4)	12	6	4	2	−351.8	38.1
6	(1,3,4)	16	4	4	0	−955.2	89.1	60	(2,2,3)	12	6	4	2	−346.5	33.1
7	(2,2,3)	16	4	4	0	−954.9	92.1	61	(1,2,5)	12	10	0	2	−345.6	30.1
8	(2,2,3)	16	2	6	0	−946.7	93.1	62	(1,3,4)	12	6	4	2	−343.7	37.1
9	(1,3,4)	16	6	2	0	−941.8	92.1	63	(1,3,4)	12	8	2	2	−328.6	35.1
10	(2,2,4)	16	0	8	0	−934.7	74.1	64	(1,1,6)	8	8	4	4	−303.9	27.1
11	(2,2,4)	16	6	2	0	−912.8	88.1	65	(2,2,3)	12	0	10	2	−288.7	14.1
12	(2,2,4)	16	4	4	0	−905.1	88.1	66	(0,2,6)	8	8	4	4	−282.0	17.1
13	(2,2,3)	16	0	8	0	−890.3	76.1	67	(0,2,6)	8	8	4	4	−278.7	17.1
14	(2,2,4)	16	4	4	0	−887.0	91.1	68	(2,2,3)	12	2	8	2	−276.3	24.1
15	(2,2,3)	16	2	6	0	−883.1	79.1	69	(2,2,3)	12	2	8	2	−275.6	16.1
16	(2,2,4)	16	4	4	0	−881.7	88.1	70	(0,2,6)	8	8	4	4	−272.8	14.1
17	(2,2,3)	16	4	4	0	−865.2	81.1	71	(2,2,3)	12	4	6	2	−270.3	20.1
18	(2,2,3)	16	6	2	0	−860.1	85.1	72	(1,3,4)	12	4	6	2	−269.9	6.1
19	(2,2,3)	16	4	4	0	−832.9	89.1	73	(0,2,6)	8	12	0	4	−263.2	21.1
20	(2,2,3)	16	2	6	0	−797.6	70.1	74	(1,3,4)	12	6	4	2	−262.3	13.1
21	(2,2,3)	16	4	4	0	−788.9	75.1	75	(2,2,3)	12	6	4	2	−261.7	25.1
22	(2,2,3)	16	6	2	0	−760.9	81.1	76	(1,3,4)	12	8	2	2	−260.2	22.1
23	(2,2,4)	16	8	0	0	−745.7	78.1	77	(2,2,4)	12	4	6	2	−237.7	16.1
24	(0,3,5)	12	4	6	2	−737.5	52.1	78	(2,2,4)	12	4	6	2	−233.3	14.1
25	(0,3,5)	12	8	2	2	−721.1	61.1	79	(1,1,6)	8	8	4	4	−230.1	15.1
26	(1,2,5)	12	4	6	2	−653.2	53.1	80	(2,2,4)	12	2	8	2	−229.2	13.1
27	(1,2,5)	12	6	4	2	−631.4	63.1	81	(2,2,4)	12	4	6	2	−224.7	6.1
28	(1,2,5)	12	4	6	2	−624.3	55.1	82	(2,2,4)	12	6	4	2	−223.1	16.1
29	(2,2,4)	12	4	6	2	−603.9	61.1	83	(2,2,4)	12	6	4	2	−219.1	15.1
30	(0,4,4)	12	6	4	2	−592.8	44.1	84	(2,2,4)	12	4	6	2	−216.5	5.1
31	(1,2,5)	12	6	4	2	−546.5	46.1	85	(1,1,6)	8	10	2	4	−213.3	24.1
32	(1,2,5)	12	6	4	2	−534.7	51.1	86	(2,2,3)	12	2	8	2	−199.8	7.1
33	(1,2,5)	12	8	2	2	−529.2	45.1	87	(2,2,4)	12	8	2	2	−195.7	15.1
34	(1,3,4)	12	6	4	2	−521.9	53.1	88	(2,2,3)	12	6	4	2	−178.4	15.1
35	(1,3,4)	12	2	8	2	−479.1	33.1	89	(2,2,3)	12	4	6	2	−169.4	5.1
36	(1,3,4)	12	4	6	2	−469.4	37.1	90	(2,2,3)	12	6	4	2	−165.6	7.1
37	(0,2,6)	8	8	4	4	−469.1	39.1	91	(1,3,4)	12	8	2	2	−162.0	7.1
38	(2,2,3)	12	2	8	2	−465.9	42.1	92	(1,3,4)	12	10	0	2	−157.9	13.1
39	(1,3,4)	12	4	6	2	−465.2	39.1	93	(2,2,3)	12	8	2	2	−150.8	12.1
40	(1,2,5)	12	4	6	2	−457.8	26.1	94	(2,2,3)	12	8	2	2	−146.4	12.1
41	(1,3,4)	12	2	8	2	−455.1	31.1	95	(1,1,6)	8	8	4	4	−144.9	2.1
42	(1,3,4)	12	4	6	2	−449.3	46.1	96	(1,2,5)	8	6	6	4	−133.4	13.1
43	(1,2,5)	12	8	2	2	−444.4	46.1	97	(0,4,4)	8	4	8	4	−103.3	1.1
44	(1,2,5)	12	6	4	2	−431.3	36.1	98	(0,1,7)	4	12	2	6	−93.6	6.1
45	(1,3,4)	12	4	6	2	−430.1	38.1	99	(0,4,4)	8	8	4	4	−85.9	6.1
46	(1,3,4)	12	6	4	2	−430.0	40.1	100	(0,4,4)	8	8	4	4	−83.4	6.1
47	(2,2,4)	12	2	8	2	−425.5	32.1	101	(2,2,3)	12	2	8	2	−83.3	−2.9
48	(2,2,4)	12	4	6	2	−411.3	41.1	102	(2,2,3)	12	6	4	2	−72.9	−0.9
49	(2,2,4)	12	6	4	2	−409.6	42.1	103	(0,3,5)	8	6	6	4	−48.2	−6.9
50	(2,2,4)	12	4	6	2	−405.7	33.1	104	(0,3,5)	8	6	6	4	−42.1	−3.9
51	(2,2,4)	12	6	4	2	−403.6	41.1	105	(0,3,5)	8	10	2	4	−36.3	−1.9
52	(2,2,4)	12	6	4	2	−403.2	34.1	106	(1,2,5)	8	6	6	4	−32.9	1.1
53	(2,2,4)	12	4	6	2	−400.4	43.1	107	(1,3,4)	8	8	4	4	−29.5	12.1
54	(2,2,4)	12	8	2	2	−387.3	37.1	108	(1,2,5)	8	6	6	4	−29.5	3.1
109	(0,3,5)	8	10	2	4	−20.0	−3.9								
110	(1,2,5)	8	8	4	4	−18.1	11.1								
111	(1,2,5)	8	8	4	4	−7.5	6.1								
112	(2,2,4)	12	10	0	2	−3.3	−10.9								
**113 ***	**(0,0,8)**	0	16	0	8	**0.0**	**0.0**								
114	(2,2,3)	12	6	4	2	28.6	−19.9								
115	(1,3,4)	8	4	8	4	31.3	−3.9								
116	(2,2,3)	12	8	2	2	33.4	−15.9								
117	(2,2,3)	8	4	8	4	36.0	−1.9								
118	(1,2,5)	8	4	8	4	39.5	−13.9								
119	(1,2,5)	8	6	6	4	42.4	−8.9								
120	(1,3,4)	8	6	6	4	44.8	−3.9								
121	(1,3,4)	8	6	6	4	51.4	−5.9								
122	(2,2,4)	8	2	10	4	67.9	−15.9								
**123 ***	**(0,4,4)**	**8**	**4**	**8**	**4**	**82.7**	**−20.9**								
124	(2,2,4)	8	4	8	4	83.7	−12.9								
125	(2,2,4)	8	4	8	4	92.8	−9.9								
126	(0,4,4)	8	8	4	4	98.5	−16.9								
127	(2,2,4)	8	8	4	4	110.9	−2.9								
128	(0,4,4)	8	12	0	4	121.7	−12.9								
129	(1,3,4)	8	4	8	4	122.8	−16.9								
130	(2,2,3)	8	4	8	4	126.2	−13.9								
131	(1,2,5)	8	4	8	4	126.4	−24.9								
132	(1,3,4)	8	6	6	4	133.6	−13.9								
133	(2,2,3)	8	4	8	4	136.4	−13.9								
134	(1,3,4)	8	6	6	4	139.8	−17.9								
135	(1,2,5)	8	8	4	4	140.5	−21.9								
136	(1,3,4)	8	6	6	4	140.9	−17.9								
137	(2,2,3)	8	6	6	4	142.5	−9.9								
138	(1,2,5)	8	6	6	4	146.3	−21.9								
139	(1,3,4)	8	8	4	4	155.6	−13.9								
140	(1,3,4)	8	2	10	4	206.9	−33.9								
141	(2,2,3)	8	2	10	4	221.0	−29.9								
142	(0,2,6)	4	10	4	6	222.3	−23.9								
143	(0,2,6)	4	10	4	6	242.3	−20.9								
144	(2,2,4)	8	4	8	4	258.9	−32.9								
145	(2,2,4)	8	0	12	4	259.6	−40.9								
146	(1,1,6)	4	8	6	6	269.6	−27.9								
147	(0,3,5)	4	8	6	6	273.1	−21.9								
148	(2,2,4)	8	6	6	4	277.4	−31.9								
149	(2,2,3)	8	2	10	4	296.2	−38.9								
150	(1,3,4)	8	2	10	4	304.0	−44.9								
151	(2,2,3)	8	4	8	4	305.1	−32.9								
152	(1,3,4)	8	4	8	4	310.5	−43.9								
153	(2,2,4)	8	0	12	4	414.6	−61.9								
154	(1,2,5)	4	6	8	6	445.0	−46.9								
155	(1,3,4)	4	6	8	6	450.6	−36.9								
156	(2,2,4)	8	4	8	4	456.8	−60.9								
157	(2,2,3)	8	0	12	4	462.3	−64.9								
158	(2,2,4)	4	4	10	6	565.5	−54.9								
159	(2,2,4)	4	6	8	6	585.1	−54.9								
160	(2,2,3)	4	4	10	6	614.9	−57.9								
161	(2,2,3)	4	4	10	6	621.4	−58.9								
162	(0,4,4)	0	8	8	8	720.0	−52.9								

**Table A5:** B3LYP set of optimized S222 cell parameters per SIC. As in Table A3, SICs are ordered by increasing energy and labelled (L${}_{\mathrm{SIC}}$). In a given SIC, the parameters can permute depending on the considered configuration.

L${}_{\mathrm{SIC}}$	CEC	a	b	c	L${}_{\mathrm{SIC}}$	CEC	a	b	c	L${}_{\mathrm{SIC}}$	CEC	a	b	c
1	(0,4,4)	8.4343	8.3081	8.3082	55	(2,3,3)	8.3685	8.3375	8.3386	109	(2,2,4)	8.3671	8.3707	8.3058
2	(0,4,4)	8.4398	8.3068	8.3068	56	(1,3,4)	8.4003	8.3417	8.3051	110	(1,2,5)	8.3990	8.3700	8.2780
3	(2,2,4)	8.3710	8.3710	8.3079	57	(2,3,3)	8.3677	8.3382	8.3419	111	(0,1,7)	8.4320	8.4000	8.2170
4	(1,3,4)	8.4020	8.3391	8.3083	58	(1,3,4)	8.4024	8.3379	8.3070	112	(1,2,5)	8.4000	8.3680	8.2770
5	(1,3,4)	8.4067	8.3383	8.3067	59	(2,2,4)	8.3710	8.3714	8.3063	113	(1,2,5)	8.4010	8.3670	8.2780
6	(2,2,4)	8.3702	8.3701	8.3083	60	(2,3,3)	8.3697	8.3360	8.3418	114	(0,3,5)	8.4340	8.3390	8.2740
7	(2,3,3)	8.3708	8.3407	8.3385	61	(1,3,4)	8.4013	8.3379	8.3088	115	(2,3,3)	8.3684	8.3406	8.3356
8	(2,3,3)	8.3700	8.3392	8.3392	62	(2,3,3)	8.3688	8.3405	8.3366	116	(1,3,4)	8.4012	8.3384	8.3057
9	(2,3,3)	8.3738	8.3383	8.3385	63	(1,3,4)	8.4016	8.3377	8.3087	117	(0,3,5)	8.4340	8.3360	8.2770
10	(1,3,4)	8.4033	8.3406	8.3069	64	(2,3,3)	8.3681	8.3390	8.3383	118	(1,2,5)	8.4010	8.3680	8.2760
11	(1,3,4)	8.4048	8.3395	8.3070	65	(2,3,3)	8.3697	8.3383	8.3381	119	(2,2,4)	8.3682	8.3696	8.3064
12	(2,2,4)	8.3707	8.3732	8.3067	66	(1,3,4)	8.4026	8.3378	8.3081	120	(2,2,4)	8.3694	8.3726	8.3043
13	(2,3,3)	8.3697	8.3390	8.3406	67	(1,3,4)	8.4009	8.3396	8.3052	121	(2,2,4)	8.3662	8.3701	8.3080
14	(2,2,4)	8.3719	8.3716	8.3071	68	(2,2,4)	8.3685	8.3694	8.3074	122	(1,3,4)	8.3993	8.3382	8.3079
15	(2,2,4)	8.3705	8.3730	8.3069	69	(1,2,5)	8.4040	8.3700	8.2760	123	(2,3,3)	8.3712	8.3371	8.3371
16	(2,2,4)	8.3733	8.3710	8.3069	70	(2,3,3)	8.3713	8.3395	8.3362	124	(0,4,4)	8.4300	8.3034	8.3108
17	(2,3,3)	8.3717	8.3383	8.3399	71	(2,3,3)	8.3696	8.3386	8.3366	125	(2,3,3)	8.3719	8.3371	8.3349
18	(2,3,3)	8.3704	8.3399	8.3399	72	(2,2,4)	8.3696	8.3696	8.3069	126	(1,3,4)	8.3997	8.3387	8.3056
19	(2,3,3)	8.3728	8.3380	8.3399	73	(1,1,6)	8.4010	8.4010	8.2460	127	(2,3,3)	8.3661	8.3390	8.3390
20	(2,3,3)	8.3717	8.3391	8.3380	74	(1,3,4)	8.4022	8.3360	8.3084	128	(1,2,5)	8.4000	8.3680	8.2770
21	(2,3,3)	8.3714	8.3406	8.3379	75	(2,2,4)	8.3697	8.3701	8.3064	129	(2,3,3)	8.3679	8.3364	8.3406
22	(0,3,5)	8.4340	8.3390	8.2760	76	(2,2,4)	8.3695	8.3708	8.3052	130	(1,3,4)	8.4011	8.3380	8.3054
23	(2,3,3)	8.3718	8.3394	8.3395	77	(0,2,6)	8.4328	8.3692	8.2463	131	(1,3,4)	8.3990	8.3383	8.3075
24	(0,3,5)	8.4370	8.3380	8.2760	78	(2,2,4)	8.3695	8.3674	8.3087	132	(1,3,4)	8.4010	8.3384	8.3053
25	(1,2,5)	8.4020	8.3700	8.2770	79	(0,2,6)	8.4335	8.3689	8.2456	133	(0,4,4)	8.4324	8.3066	8.3067
26	(1,2,5)	8.4020	8.3700	8.2770	80	(2,2,4)	8.3690	8.3711	8.3065	134	(2,2,4)	8.3654	8.3695	8.3111
27	(2,2,4)	8.3707	8.3706	8.3075	81	(0,2,6)	8.4327	8.3692	8.2461	135	(1,3,4)	8.3980	8.3386	8.3059
28	(2,2,4)	8.3727	8.3727	8.3063	82	(2,2,4)	8.3709	8.3691	8.3067	136	(2,3,3)	8.3686	8.3367	8.3371
29	(1,2,5)	8.4030	8.3700	8.2760	83	(1,3,4)	8.4020	8.3374	8.3085	137	(1,2,5)	8.4000	8.3680	8.2780
30	(0,4,4)	8.4347	8.3053	8.3088	84	(2,3,3)	8.3677	8.3396	8.3379	138	(1,2,5)	8.4010	8.3710	8.2740
31	(1,2,5)	8.4030	8.3700	8.2760	85	(1,1,6)	8.4010	8.4010	8.2460	139	(2,2,4)	8.3668	8.3669	8.3076
32	(1,3,4)	8.4017	8.3373	8.3077	86	(2,3,3)	8.3707	8.3396	8.3360	140	(1,3,4)	8.4024	8.3342	8.3087
33	(2,3,3)	8.3705	8.3373	8.3391	87	(2,3,3)	8.3722	8.3375	8.3362	**141**	**(0,0,8)**	**8.4315**	**8.4315**	**8.1876**
34	(1,2,5)	8.4020	8.3710	8.2760	88	(2,2,4)	8.3689	8.3706	8.3079	142	(0,4,4)	8.4346	8.3027	8.3096
35	(1,3,4)	8.4034	8.3383	8.3072	89	(0,2,6)	8.4353	8.3687	8.2456	143	(2,2,4)	8.3679	8.3700	8.3052
36	(1,3,4)	8.4000	8.3391	8.3075	90	(1,1,6)	8.4010	8.4020	8.2460	144	(2,3,3)	8.3683	8.3387	8.3349
37	(1,3,4)	8.4032	8.3366	8.3076	91	(2,3,3)	8.3684	8.3397	8.3361	145	(1,3,4)	8.3979	8.3347	8.3092
38	(1,2,5)	8.4040	8.3700	8.2750	92	(1,2,5)	8.4000	8.3700	8.2770	146	(2,3,3)	8.3661	8.3364	8.3403
39	(1,3,4)	8.4013	8.3388	8.3076	93	(1,3,4)	8.4018	8.3373	8.3079	147	(2,2,4)	8.3689	8.3675	8.3073
40	(2,2,4)	8.3682	8.3707	8.3075	94	(2,3,3)	8.3704	8.3390	8.3375	148	(0,2,6)	8.4307	8.3706	8.2448
41	(1,2,5)	8.4010	8.3700	8.2760	95	(2,3,3)	8.3684	8.3409	8.3376	149	(1,3,4)	8.3992	8.3378	8.3055
42	(1,3,4)	8.4003	8.3383	8.3093	96	(0,4,4)	8.4302	8.3077	8.3078	150	(1,1,6)	8.3990	8.3990	8.2470
43	(2,3,3)	8.3701	8.3375	8.3375	97	(1,1,6)	8.4000	8.4000	8.2460	151	(0,2,6)	8.4306	8.3673	8.2484
44	(1,3,4)	8.4019	8.3364	8.3092	98	(1,3,4)	8.4031	8.3402	8.3044	152	(0,3,5)	8.4300	8.3390	8.2770
45	(2,2,4)	8.3685	8.3696	8.3096	99	(2,3,3)	8.3709	8.3391	8.3356	153	(2,2,4)	8.3662	8.3662	8.3077
46	(2,2,4)	8.3681	8.3721	8.3069	100	(0,4,4)	8.4327	8.3072	8.3071	154	(2,3,3)	8.3662	8.3369	8.3369
47	(2,2,4)	8.3697	8.3705	8.3074	101	(0,4,4)	8.4325	8.3072	8.3073	155	(2,2,4)	8.3655	8.3655	8.3106
48	(1,3,4)	8.4032	8.3398	8.3052	102	(0,3,5)	8.4320	8.3360	8.2780	156	(1,2,5)	8.3980	8.3700	8.2750
49	(1,2,5)	8.4010	8.3720	8.2760	103	(0,3,5)	8.4320	8.3400	8.2740	157	(1,3,4)	8.3961	8.3392	8.3083
50	(0,2,6)	8.4337	8.3699	8.2461	104	(1,3,4)	8.4000	8.3387	8.3077	158	(2,2,4)	8.3685	8.3685	8.3045
51	(2,2,4)	8.3695	8.3696	8.3090	105	(1,2,5)	8.4000	8.3710	8.2750	159	(2,2,4)	8.3643	8.3719	8.3061
52	(2,2,4)	8.3683	8.3748	8.3047	106	(1,2,5)	8.4030	8.3680	8.2760	160	(2,3,3)	8.3705	8.3354	8.3354
53	(1,2,5)	8.4030	8.3710	8.2760	107	(2,3,3)	8.3666	8.3391	8.3393	161	(2,3,3)	8.3663	8.3375	8.3375
54	(2,2,4)	8.3699	8.3694	8.3091	108	(1,3,4)	8.3980	8.3388	8.3081	162	(0,4,4)	8.4261	8.3087	8.3087

**Table A6:** Total energy E${}_{T}$ (in E${}_{h}$) and volume V (in Å${}^{3}$) for the 39 SICs of the (2,3,3) CEC of the 2 × 2 × 2 supercell (S222) at PBE0 level for the FM solution and for 2 formula units of KFeF${}_{3}$. The SIC multiplicity is given by the M column. ΔE (in μE${}_{h}$) and ΔV (in mÅ${}^{3}$) are the energy and volume differences (or relative energy or volume) with respect to the REF case. In the last three columns the lattice parameters of the S222 supercell (40 atoms) are shown.

I	E${}_{T}$	ΔE	M	VOL	ΔV	a	b	c
	−4325.748339	0.0	1	142.8361	0.0	8.1502	8.3727	8.3727
1	−4325.749389	−1049.6	48	142.8489	12.8	8.3169	8.2896	8.2879
2	−4325.749358	−1018.9	48	142.8582	22.0	8.3194	8.2877	8.2878
3	−4325.749352	−1013.4	24	142.8255	−10.6	8.3164	8.2883	8.2883
4	−4325.749318	−979.2	48	142.8404	4.3	8.3165	8.2879	8.2895
5	−4325.749280	−940.7	24	142.8517	15.6	8.3164	8.2891	8.2891
6	−4325.749276	−937.4	48	142.8495	13.4	8.3177	8.2876	8.2891
7	−4325.749253	−913.7	48	142.8622	26.1	8.3190	8.2873	8.2889
8	−4325.749252	−912.6	48	142.8323	−3.9	8.3179	8.2880	8.2875
9	−4325.749213	−874.2	48	142.8475	11.4	8.3175	8.2892	8.2876
10	−4325.749177	−838.2	48	142.8595	23.4	8.3175	8.2886	8.2888
11	−4325.748950	−611.0	48	142.8081	−28.0	8.3168	8.2867	8.2885
12	−4325.748895	−556.3	24	142.7824	−53.7	8.3170	8.2868	8.2868
13	−4325.748824	−485.4	48	142.7752	−61.0	8.3152	8.2868	8.2881
14	−4325.748808	−468.8	48	142.8251	−11.0	8.3146	8.2875	8.2909
15	−4325.748802	−463.5	48	142.7911	−45.0	8.3151	8.2897	8.2862
16	−4325.748796	−457.1	48	142.8214	−14.8	8.3162	8.2858	8.2908
17	−4325.748782	−443.1	48	142.7892	−46.9	8.3149	8.2880	8.2880
18	−4325.748758	−419.0	48	142.8007	−35.4	8.3161	8.2880	8.2875
19	−4325.748727	−388.2	48	142.8155	−20.6	8.3175	8.2890	8.2859
20	−4325.748723	−383.8	48	142.7786	−57.6	8.3161	8.2880	8.2862
21	−4325.748665	−326.2	48	142.7891	−47.0	8.3146	8.2890	8.2873
22	−4325.748652	−313.5	48	142.8060	−30.1	8.3170	8.2891	8.2858
23	−4325.748641	−302.0	48	142.8011	−35.0	8.3187	8.2870	8.2859
24	−4325.748633	−293.8	48	142.7710	−65.1	8.3148	8.2891	8.2859
25	−4325.748606	−266.9	48	142.8158	−20.3	8.3169	8.2885	8.2870
26	−4325.748602	−263.5	48	142.8154	−20.7	8.3153	8.2900	8.2870
27	−4325.748564	−224.9	48	142.7953	−40.9	8.3171	8.2886	8.2856
28	−4325.748488	−148.5	48	142.7836	−52.5	8.3135	8.2886	8.2884
29	−4325.748474	−135.4	48	142.7823	−53.8	8.3153	8.2898	8.2855
30	−4325.748445	−106.5	24	142.7945	−41.6	8.3179	8.2867	8.2867
31	−4325.748412	−72.7	48	142.7701	−66.1	8.3183	8.2868	8.2848
32	−4325.748402	−63.2	24	142.7709	−65.2	8.3132	8.2883	8.2883
33	−4325.748367	−28.3	48	142.7847	−51.4	8.3147	8.2863	8.2897
34	−4325.748351	−12.1	48	142.7483	−87.9	8.3151	8.2865	8.2869
35	−4325.748279	60.0	48	142.7415	−94.6	8.3153	8.2881	8.2848
36	−4325.748249	89.9	48	142.7558	−80.3	8.3131	8.2895	8.2864
37	−4325.748165	173.5	24	142.7112	−124.9	8.3131	8.2866	8.2866
38	−4325.747976	363.2	24	142.7349	−101.2	8.3170	8.2854	8.2854
39	−4325.747968	370.8	24	142.7340	−102.1	8.3136	8.2870	8.2870

**Table A7:** As Table A6, for HSE06.

I	E${}_{T}$	ΔE	M	VOL	ΔV	a	b	c
	−4325.741347	0.0	1	142.8354	0.0	8.1489	8.3733	8.3733
1	−4325.742425	−1077.7	48	142.8448	9.4	8.3169	8.2877	8.2895
2	−4325.742392	−1044.5	48	142.8545	19.1	8.3197	8.2874	8.2876
3	−4325.742390	−1042.4	24	142.8201	−15.3	8.3165	8.2881	8.2881
4	−4325.742352	−1004.4	48	142.8361	0.7	8.3165	8.2877	8.2894
5	−4325.742317	−969.7	24	142.8468	11.4	8.3163	8.2890	8.2890
6	−4325.742309	−961.5	48	142.8461	10.7	8.3178	8.2874	8.2890
7	−4325.742284	−936.9	48	142.8278	−7.6	8.3180	8.2878	8.2874
8	−4325.742282	−934.9	48	142.8599	24.5	8.3192	8.2870	8.2888
9	−4325.742242	−895.1	48	142.8434	8.0	8.3176	8.2891	8.2873
10	−4325.742205	−857.4	48	142.8572	21.8	8.3176	8.2885	8.2888
11	−4325.741986	−638.6	48	142.8025	−32.8	8.3170	8.2864	8.2883
12	−4325.741931	−583.8	24	142.7752	−60.2	8.3170	8.2866	8.2866
13	−4325.741861	−513.8	48	142.7682	−67.2	8.3152	8.2866	8.2879
14	−4325.741837	−489.9	48	142.8212	−14.2	8.3145	8.2873	8.2909
15	−4325.741835	−488.1	48	142.7849	−50.5	8.3151	8.2896	8.2860
16	−4325.741824	−476.5	48	142.8171	−18.3	8.3163	8.2854	8.2908
17	−4325.741814	−466.9	48	142.7832	−52.1	8.3148	8.2879	8.2878
18	−4325.741788	−440.3	48	142.7957	−39.7	8.3163	8.2877	8.2873
19	−4325.741755	−407.4	48	142.7723	−63.1	8.3162	8.2878	8.2859
20	−4325.741753	−406.1	48	142.8112	−24.1	8.3177	8.2889	8.2856
21	−4325.741695	−348.0	48	142.7819	−53.4	8.3145	8.2888	8.2872
22	−4325.741678	−330.8	48	142.8016	−33.8	8.3172	8.2889	8.2855
23	−4325.741667	−320.0	48	142.7965	−38.9	8.3189	8.2868	8.2856
24	−4325.741665	−317.9	48	142.7643	−71.1	8.3147	8.2890	8.2857
25	−4325.741628	−280.9	48	142.8126	−22.8	8.3170	8.2883	8.2869
26	−4325.741626	−278.6	48	142.8121	−23.3	8.3153	8.2900	8.2869
27	−4325.741589	−241.4	48	142.7909	−44.5	8.3172	8.2885	8.2853
28	−4325.741518	−170.8	48	142.7782	−57.2	8.3134	8.2883	8.2885
29	−4325.741501	−153.5	48	142.7776	−57.7	8.3153	8.2897	8.2852
30	−4325.741467	−120.1	24	142.7907	−44.7	8.3180	8.2865	8.2865
31	−4325.741442	−95.0	48	142.7632	−72.2	8.3185	8.2865	8.2845
32	−4325.741432	−84.4	24	142.7649	−70.5	8.3131	8.2882	8.2882
33	−4325.741394	−46.7	48	142.7791	−56.3	8.3147	8.2859	8.2897
34	−4325.741385	−37.3	48	142.7404	−95.0	8.3152	8.2862	8.2866
35	−4325.741311	36.0	48	142.7342	−101.2	8.3152	8.2880	8.2845
36	−4325.741278	69.6	48	142.7494	−86.0	8.3130	8.2861	8.2895
37	−4325.741200	147.6	24	142.7030	−132.4	8.3131	8.2864	8.2864
38	−4325.741006	341.3	24	142.7276	−107.8	8.3172	8.2851	8.2851
39	−4325.740999	348.2	24	142.7261	−109.3	8.3135	8.2869	8.2869

**Table A8:** As Table A6, for UHF.

I	E${}_{T}$	ΔE	M	VOL	ΔV	a	b	c
	−4320.657779	0.0	1	148.3826	0.0	8.2509	8.4815	8.4815
1	−4320.658161	−381.9	48	148.4167	34.1	8.4235	8.3956	8.3946
2	−4320.658154	−375.3	48	148.4207	38.1	8.4240	8.3955	8.3944
3	−4320.658142	−362.8	48	148.4322	49.6	8.4264	8.3944	8.3937
4	−4320.658135	−356.5	24	148.4331	50.5	8.4253	8.3941	8.3953
5	−4320.658135	−350.1	24	148.4331	50.5	8.4251	8.3940	8.3950
6	−4320.658082	−303.6	48	148.4274	44.7	8.4265	8.3939	8.3939
7	−4320.658042	−263.5	48	148.4240	41.3	8.4251	8.3956	8.3934
8	−4320.658041	−262.3	48	148.4278	45.2	8.4266	8.3940	8.3937
9	−4320.658006	−227.4	48	148.4342	51.6	8.4260	8.3950	8.3937
10	−4320.657965	−186.5	48	148.4276	45.0	8.4248	8.3963	8.3932
11	−4320.657868	−89.1	24	148.3641	−18.5	8.4235	8.3936	8.3936
12	−4320.657859	−80.7	48	148.3874	4.8	8.4245	8.3937	8.3938
13	−4320.657817	−38.0	48	148.3627	−19.9	8.4232	8.3938	8.3936
14	−4320.657803	−23.8	48	148.3883	5.6	8.4244	8.3935	8.3942
15	−4320.657797	−18.6	48	148.3855	2.9	8.4245	8.3933	8.3941
16	−4320.657764	15.2	48	148.3655	−17.1	8.4232	8.3942	8.3933
17	−4320.657763	15.5	48	148.3629	−19.8	8.4233	8.3942	8.3932
18	−4320.657762	17.2	48	148.3656	−17.0	8.4231	8.3942	8.3935
19	−4320.657757	21.8	48	148.3596	−23.1	8.4239	8.3932	8.3934
20	−4320.657752	26.4	48	148.3810	−1.7	8.4246	8.3939	8.3932
21	−4320.657745	33.6	48	148.3733	−9.3	8.4244	8.3940	8.3928
22	−4320.657743	35.8	48	148.3748	−7.8	8.4244	8.3940	8.3929
23	−4320.657739	39.8	48	148.3673	−15.4	8.4228	8.3946	8.3935
24	−4320.657709	69.3	48	148.3551	−27.5	8.4235	8.3938	8.3929
25	−4320.657681	97.9	48	148.3612	−21.4	8.4226	8.3951	8.3928
26	−4320.657680	98.5	48	148.3647	−18.0	8.4240	8.3939	8.3929
27	−4320.657653	126.0	24	148.3484	−34.2	8.4237	8.3930	8.3930
28	−4320.657646	132.9	48	148.3443	−38.4	8.4226	8.3939	8.3929
29	−4320.657632	147.1	48	148.3563	−26.3	8.4243	8.3935	8.3925
30	−4320.657523	256.1	48	148.3436	−39.1	8.4225	8.3934	8.3936
31	−4320.657517	261.6	48	148.3377	−45.0	8.4233	8.3935	8.3924
32	−4320.657514	264.4	24	148.3389	−43.8	8.4223	8.3935	8.3935
33	−4320.657509	270.1	48	148.3579	−24.7	8.4236	8.3933	8.3935
34	−4320.657456	322.6	48	148.3264	−56.2	8.4224	8.3930	8.3931
35	−4320.657411	367.9	48	148.3247	−57.9	8.4219	8.3931	8.3935
36	−4320.657402	376.7	48	148.3188	−63.8	8.4230	8.3931	8.3920
37	−4320.657290	488.3	24	148.3002	−82.4	8.4215	8.3928	8.3928
38	−4320.657173	605.8	24	148.3049	−77.7	8.4225	8.3924	8.3924
39	−4320.657169	609.9	24	148.3051	−77.5	8.4222	8.3926	8.3926
